# Pelvic rotation correction combined with Schroth exercises for pelvic and spinal deformities in mild adolescent idiopathic scoliosis: A randomized controlled trial

**DOI:** 10.1371/journal.pone.0307955

**Published:** 2024-07-30

**Authors:** Yafei Zhang, Tingting Chai, Hao Weng, Yang Liu

**Affiliations:** Department of Rehabilitation Medicine, Aerospace Center Hospital, Beijing, China; New York University College of Dentistry, UNITED STATES OF AMERICA

## Abstract

**Introduction:**

Individualized treatment of spinal deformity is needed for adolescent idiopathic scoliosis (AIS), and the integration of pelvic rotation correction based on proprioceptive neuromuscular facilitation (PNF) into regular physiotherapy may be a promising approach. However, few high-quality studies have investigated its effects. This study aimed to evaluate the efficacy of pelvic rotation correction combined with Schroth exercises in the treatment of mild AIS.

**Methods:**

This was a randomized controlled trial. Forty-two AIS patients were randomly divided into experimental and control groups. Both groups underwent 20 therapeutic sessions over 24 weeks. All patients (n = 42) performed Schroth exercises at each session. In addition, the experimental group (n = 21) also participated in a pelvic rotation correction program based on PNF at each session. The primary outcome was the concave/convex ratio of hipbone widths, and the secondary outcomes included the Cobb angle, trunk rotation angle, self-perception, apical vertebral translation, and apical vertebral rotation. Patients were evaluated before and after 24 weeks of intervention.

**Results:**

There was a significant between-group difference in the change from baseline between the experimental and control groups for the following parameters: concave/convex ratio 2.89% (95% confidence interval [CI], 1.58 to 4.20, *P*<0.001), trunk rotation angle −1.26° (95% CI, −2.20 to −0.32; *P* = 0.01), and apical vertebral rotation improved by at least one class from baseline in 3 patients (14.3%) in the control group and 9 patients (42.9%) in the experimental group (*P* = 0.04). While Cobb angle −1.60° (95% CI, −7.75 to 0.54; *P* = 0.14), self-image 0.149 (95% CI, 0.001 to 0.297; *P* = 0.049), apical vertebral translation −0.58 mm (95% CI, −3.83 to 2.67; *P* = 0.72), and pelvic obliquity 0.10° (95% CI, −0.21 to 0.41; *P* = 0.52) did not differ significantly.

**Conclusions:**

Pelvic rotation correction combined with Schroth exercises more effectively improved pelvic axial rotation and other spinal deformities, including trunk rotation and apical vertebral rotation, than Schroth exercises alone in the treatment of mild AIS.

## Introduction

Adolescent idiopathic scoliosis (AIS) is a three-dimensional deformity of the spine that causes aesthetic deformities during growth [1, 2]. AIS is defined as a lateral curvature of the spine of 10 degrees or more on a coronal radiograph while the patient is standing [3]. It has obvious influences on the anatomical and functional characteristics of the spine and pelvis, and the primary goal of conservative treatment is to stop curve progression in AIS patients [2]. As the main treatment for mild AIS (i.e., curves less than 25°), scoliosis-specific physiotherapy, such as Schroth exercises, has been shown to be effective in controlling scoliosis progression [4–6]. The Schroth treatment program consists of active trunk correction to realign the spine in three dimensions [7, 8]. Although the Schroth method has shown positive results in slowing curve progression [9], improving the Cobb angle [10–12], and improving the trunk rotation angle [12], there has been little evidence of its efficacy in improving pelvic rotation.

The pelvis has been described as a "pelvic vertebra" in that it serves as part of the overall balance chain in the scoliotic spine [13–15]. Similar to the thoracic or lumbar vertebra in patients with scoliosis, the pelvis also has axial rotation to some extent in patients with AIS [16–20], which manifests as asymmetrical shadows on the concave and convex sides of the pelvis on posteroanterior radiographs [21]. Pelvic parameters can accurately reflect the clinical status of the patient [22, 23], while pelvic axial rotation (PAR) is a fundamental pelvic parameter that is associated with three-dimensional spinal deformities such as thoracic or thoracolumbar/lumbar apex vertebral rotation [16, 19, 24] and the Cobb angle [17, 25]. PAR also plays an essential role in overall static and dynamic postural balance and stability; axial rotation of the pelvis could alter the patterns of static trunk alignment and the dynamic mechanics of trunk-pelvis movements [26–31]. Furthermore, pelvic rotational asymmetry is associated with decreased postural stability which may predispose AIS patients to low back pain [32, 33]. Besides, in severe AIS, predicting the relationship between spinal alignment and pelvic axial plane position is critical for optimal planning of spinal deformity surgery. Qiu XS et al. [18] showed that AIS patients with preoperative asymmetrical pelvic axial rotation probably had a greater risk of coronal decompensation postoperatively. Scott L et al. [25] claimed that pelvic rotation would affect three-dimensional spinal alignment measurements in the preoperative assessment of AIS. Therefore, in the clinical management of AIS, it is evident that each patient requires individualized treatment of the spinal deformity, and incorporating pelvic rotation correction into regular physiotherapy for spinal deformity may help to achieve a more effective treatment plan for AIS.

Proprioceptive neuromuscular facilitation (PNF) technique involves movement patterns based primarily on rotational movement performed in a diagonal plane; movements practiced according to PNF are smooth and harmonized with natural muscle action, allowing for full stretching of muscle fibers during contraction [34–37]. In addition, early reports of PNF technique for trunk and pelvic symmetry in patients with AIS showed promising results, as this method allows the use of appropriate pelvic patterns to improve the spatial alignment of the pelvis and trunk in patients with AIS. Stępień et al. [38] applied the PNF technique to the pelvic girdle of adolescent girls with mild to moderate scoliosis and reported immediate improvements in trunk and pelvic deformities in the transverse plane, but the lack of radiographic evaluation and a control group limited the ability to broadly apply the study’s findings to clinical practice. Beyond that, there is little high-level evidence supporting the potential benefits of pelvic rotation correction.

We hypothesized that the integration of pelvic rotation correction based on PNF combined with Schroth exercises in the treatment of AIS would improve pelvic and spinal deformities. Therefore, we conducted this randomized controlled trial to evaluate the effect of a combined (PNF-based pelvic rotation correction exercise program added to the Schroth exercise) training program compared to the Schroth exercise alone on pelvic axial rotation and its effect on spinal and trunk deformities in the axial and coronal planes and on self-image acceptability in adolescents with mild idiopathic scoliosis.

## Materials and methods

### Design and patients

**Fig 1:**
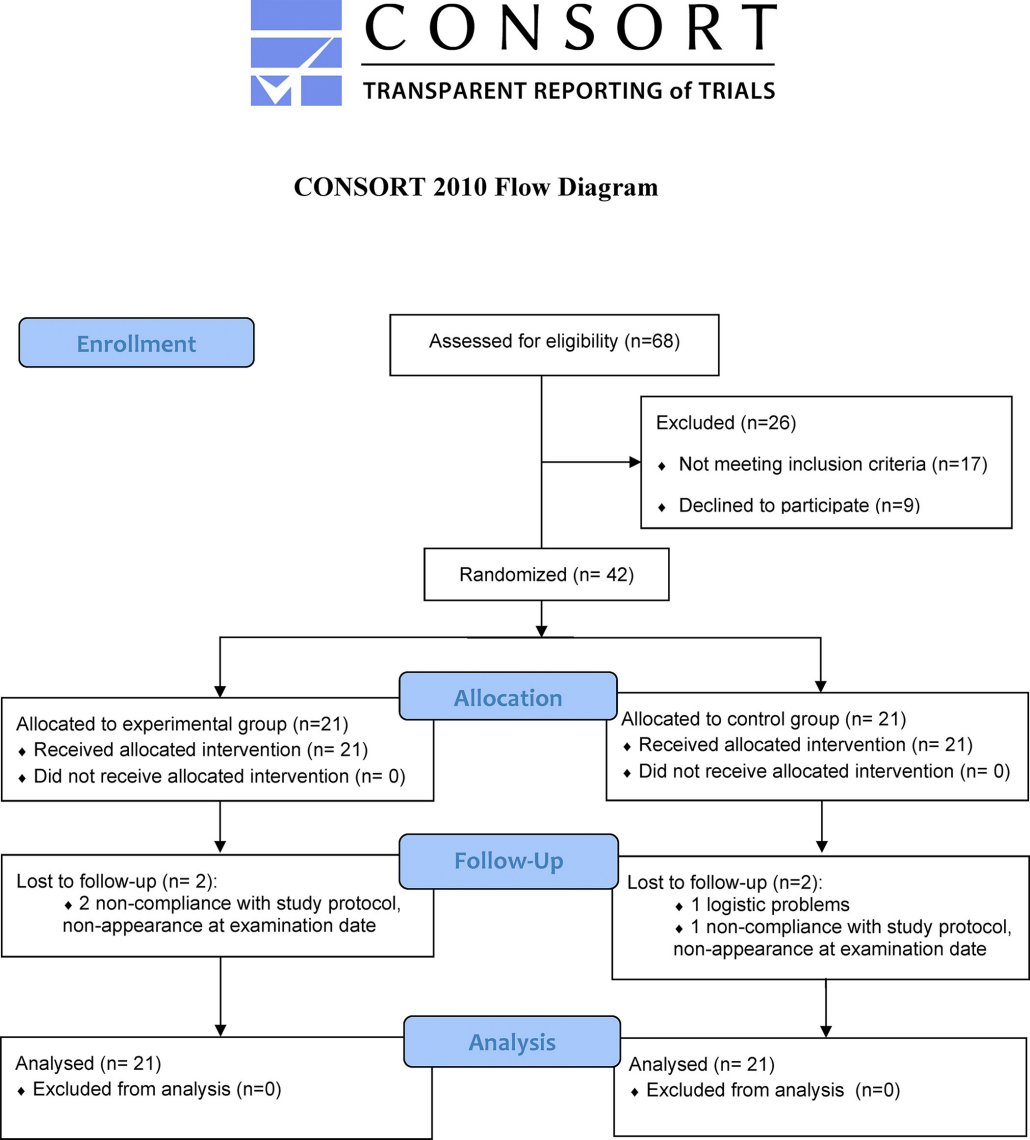
Consolidated Standards of Reporting Trials (CONSORT) 2010 flow diagram.

This is a parallel-group, assessor-blinded, randomized controlled trial. It has been carried out according to the recommendations of the Consolidated Standards of Reporting Trials (CONSORT) statements (Fig 1). The trial was conducted in accordance with the Helsinki Declaration, and approved by the Medical Ethics Committee of Aerospace Center Hospital (JAH-ER-2022-001). The recruitment period was from February 18, 2022 to November 10, 2022. Written informed consent was obtained from all study subjects and their parents or guardians prior to enrollment. The study protocol was registered at ClinicalTrials.gov (NCT05259956).

Participants with newly diagnosed adolescent idiopathic scoliosis were consecutively recruited from the rehabilitation medicine outpatient clinic of the Aerospace Center Hospital in Beijing by one experienced rehabilitation physician. The inclusion criteria were as follows: 10–18 years old, both sexes, Cobb angle 10–25°, Risser 0–5 (all skeletal maturities), and ability to travel to 24-week clinic visits. Exclusion criteria were: non-idiopathic scoliosis, including neuromuscular scoliosis, neurological scoliosis, congenital malformation, or trauma-related comorbidity; mental problems or other contraindications to exercise; previous fractures or history of operation involving the spine or lower extremities; rheumatic diseases; previous or current brace treatment or planning surgery for scoliosis.

### Randomization and blinding

After an initial exam confirming the eligibility and collecting baseline data, patients were randomly allocated in a 1:1 ratio to either the experimental group or the control group using a computerized random number function in Microsoft Excel generated by the statistician. A block randomization procedure with a random block length (block size of four and six) was employed. The random allocation sequence was transferred to a sequence of consecutively numbered, sealed, opaque envelopes, which were stored in a locked drawer until needed. When each participant was formally enrolled in the study, the sealed envelope with the assigned number was opened in front of the participant and the arm allocation was known.

The physicians who collected and assessed the outcome data and the statistician who analyzed the data were both blinded to the assigned treatment. Physiotherapists and patients could not be blinded when offering or receiving treatments. However, participants were asked not to reveal their group allocation to ensure blinding of the assessors.

### Interventions

All participants were scheduled to perform 2 sessions of training exercises per week for the first 5 weeks after baseline, followed by 1 session biweekly until the 24th week. The physiotherapist supervised all 20 sessions for each participant.

#### Control group

Each session consisted of 60 minutes of Schroth exercises [39]. The Schroth program includes the Schroth-specific exercise (self-elongation, side-shift of thorax or lumbar, shoulder corrections, de-rotation with corrective breathing and pelvic correlations) to open up the curvatures of the thoracic and lumbar spine, and the postural re-education (the correct sitting position, standing position and standing position while leaning against something) to incorporate postural corrections into activities of daily livings. The Schroth program is presented in S1 File.

The exercises were demonstrated and supervised by a certified and experienced physiotherapist, who is available to meet with each patient to monitor the learning process.

#### Experimental group

**Fig 2:**
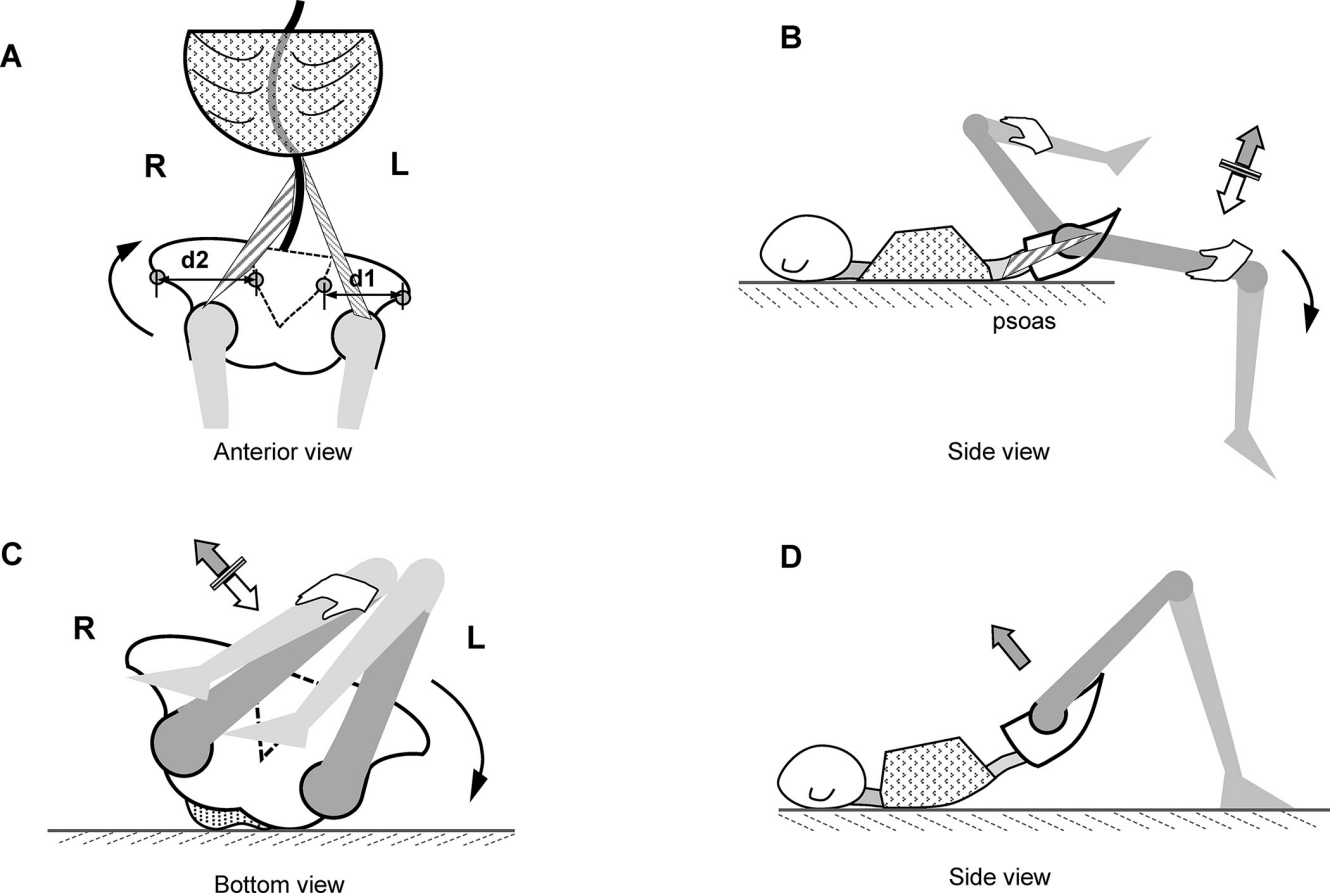
Training procedure for PNF stretching. A. Schematic illustration describing the definition of the right PAR (pelvic rotating clockwise in cephalad view). d1/d2 shows the distance of the line connecting the ipsilateral ASIS and SI. **B. The stretch of the right tight psoas.** Lying in a supine position, the patient’s right leg is dropped off the table. The patient tries to flex the hip against resistance from the therapist pushing down the knee. The white arrow represents the direction of force from the therapist; the gray arrow represents the direction of force from the patient. PNF, proprioceptive neuromuscular facilitation; PAR, pelvic axial rotation; ASIS, anterior superior iliac spine; SI, inferior ilium at the sacroiliac joint. **C. The de-rotation stretches of the pelvic girdle.** To treat a right PAR, the patient lies in a supine position with the therapist standing on the left side. The lower limbs are flexed and pulled toward the left chest until the sacral bone is lifted. **D. Active motor control exercise.** The patient lifts the pelvis and remains level while lying in the supine position.

Each session consisted of 60 minutes of Schroth-specific exercises, postural re-education (same as the control group), and a 30-minute corrective exercise program for pelvic rotation, which followed a three-step treatment procedure (Fig 2).

First, it started with a ten-minute stretch of shortened muscles of the pelvis. In order to stabilize and balance the trunk and the pelvis there should be an optimal interaction of the muscles of the abdomen, the lateral trunk, the back and the flexors and extensors of the hip, if that balance is disrupted, muscular imbalance and a weakening or shortening of the muscles involved can occur [40], scoliosis is one of the situations that break the balance. In the present study, a series of orthopedic physical tests were used to assess the imbalance of the trunk and pelvic-femoral muscles [41]. These tests determined which muscles were shortened or weakened, the target muscle groups included the hip flexors, hip extenders, hip adductors, hip abductors, lateral rotators, medial rotators, and quadratus lumborum. The PNF stretching technique was then applied to correct the muscle imbalance, where the short and tight muscles were identified and lengthened, and the weak and inhibited muscles were encouraged towards enhanced tone, strength, and stamina [42]. See part I. Stretch of asymmetric muscles of the pelvis in S2 File.

We took the right psoas (a hip flexor) as an example (Fig 2A). The Thomas test was used to detect shortened psoas. The patient lay supine while a physiotherapist flexed one of the patient’s hips, bringing the knee to the chest to flatten the lumbar spine and stabilize the pelvis. The patient held the flexed hip against the chest. If there were shortened hip flexors, the patient’s straight leg rose off the table, resulting in a muscle-stretched sensation. Then the “hold–relax” technique was used to stretch the tight psoas; it involved an isometric contraction of the psoas resisted by the physiotherapist, and the contraction was held for 3–5 seconds, followed by a 10-second further stretch by the physiotherapist to increase the range of hip extension (Fig 2B). The whole process was repeated three to four times.

Second, a ten-minute de-rotation of the pelvic girdle proceeded with the use of a modified “hold-relax” PNF stretching technique [38]. The direction of stretch was determined based on the results of the patient’s posterior-anterior radiograph. If it indicated a right PAR (pelvic rotating clockwise, in cephalad view) (Fig 2A), a left axial rotation stretch was applied with a modified bilateral lower limb pattern (flexion to the left and extension to the right). The “hold–relax” technique was performed by the patient statically contracting muscle groups of the pelvis against a resistance provided by the physiotherapist. The contraction was held for 3–5 seconds, followed by a 10-second further stretch to increase the range of left axial rotation (Fig 2C). The whole process was repeated three to four times. The treatment principle for the left PAR (counter-clockwise, in cephalad view) was the same.

Finally, ten minutes of motor control exercises focusing on enhancing spinopelvic alignment and kinetic stability were performed (Fig 2D) [43]. See S2 File for details of the exercises that were used.

### Outcome measures

All radiologic outcomes, including concave/convex ratio, Cobb angle, apical vertebral rotation, apical vertebral translation, and pelvic obliquity were measured using Image-Pro Plus software (version 6.0, Media Cybernetics, Inc., USA) on standing posterior-anterior full-spine radiographs by a senior physician. All radiographic and anthropometric measurements were obtained and analyzed in a blinded manner. Radiographic indices were measured by a senior physician and anthropometric parameters (e.g., angle of trunk rotation) by a physiotherapist. Quantitative data, including the concave/convex ratio of hipbone widths, Cobb angle of the major curve, apical vertebral translation, pelvic obliquity, and angle of trunk rotation, were measured twice, and the average of the two measurements was used. Qualitative data, such as apical vertebral rotation, were measured twice by the senior physician and reviewed by another senior physician, and if the results were consistent between the two measurements, the result was used; otherwise, the results were reevaluated by the senior physician to determine the final results. Outcome measures were assessed at baseline and week 24 of treatment. Adverse events and patient complaints during treatment were documented.

#### Primary outcome

**Fig 3:**
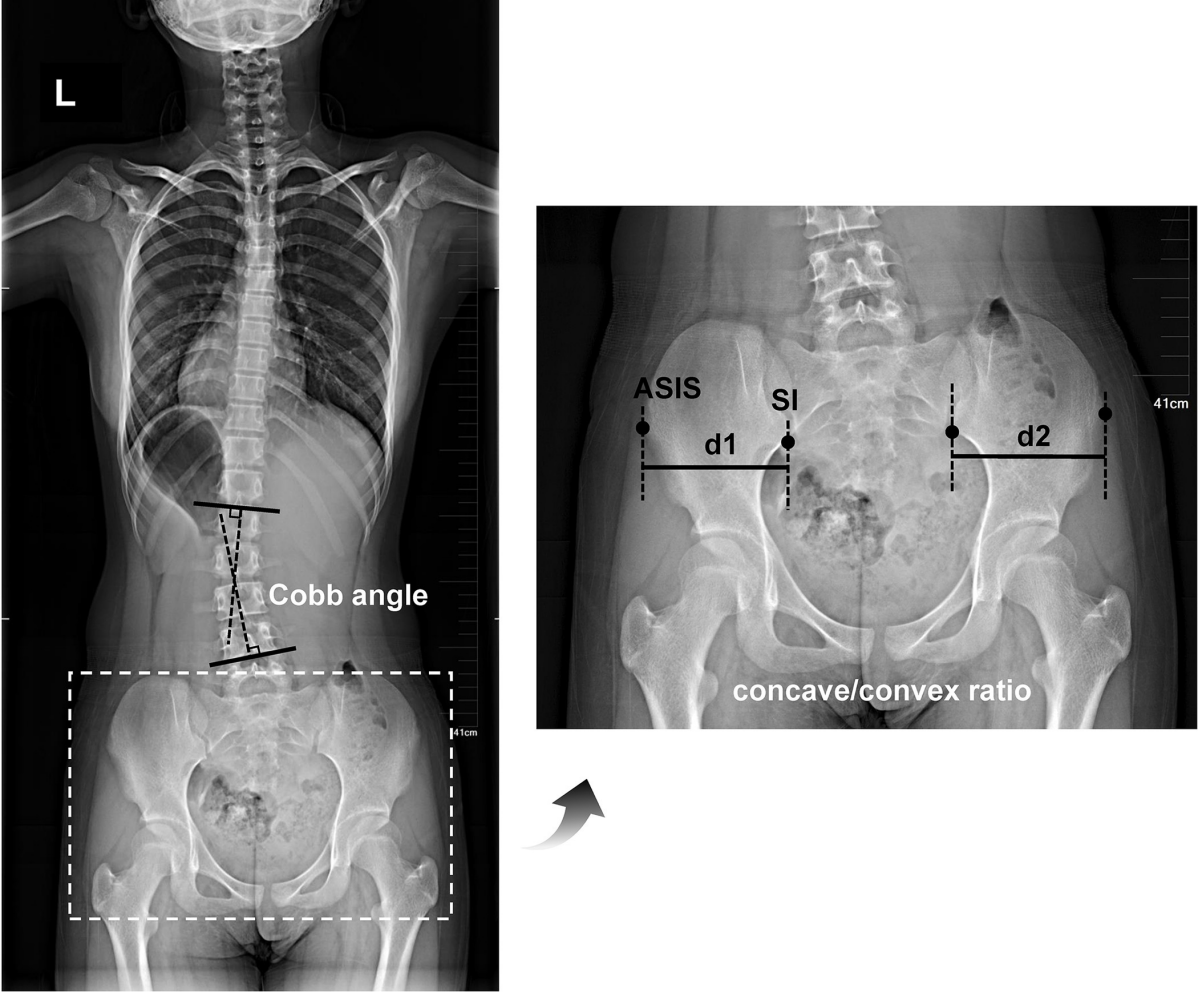
Schematic illustration describing the measurements of the Cobb angle and the concave/convex ratio of the hipbone width. An 11-year-old girl with left lumbar scoliosis, a main lumbar Cobb angle of 18°, and a concave/convex ratio (d1/d2) of 0.94. ASIS, anterior superior iliac spine; SI, sacroiliac joint.

The primary outcome was the change from baseline to week 24 in the concave/convex ratio. The concave/convex ratio of hipbone widths was calculated with the linear distance between upright lines through the inferior ilium at the sacroiliac joint (SI) and the anterior superior iliac spine (ASIS) on the left (d1) and right (d2) sides (Fig 3), and the concave/convex ratio equaled d1/d2 or d2/d1. The value of the concave/convex ratio ranged from 0 to 1, with smaller values representing a greater magnitude of pelvic axial rotation. The direction of pelvic axial rotation was the same as the wider (convex) side of the hipbone [16, 21].

#### Secondary outcomes

The secondary outcomes included the change from baseline to week 24 in the Cobb angle, angle of trunk rotation (ATR), Scoliosis Research Society (SRS-22) instrument self-image domain, apical vertebral rotation, apical vertebral translation, and pelvic obliquity.

The Cobb angle of the major curve was determined by drawing a horizontal line at the superior border of the superior-end vertebra of the major curve and another horizontal line at the inferior border of the inferior-end vertebra. Perpendicular lines were then drawn from each of the horizontal lines, the intersecting angle was determined as the Cobb angle [44], and a greater Cobb angle indicates a higher severity of scoliosis (Fig 3).

The angle of trunk rotation was measured with a Scoliometer by a physiotherapist, and the readings were obtained at a standing forward bending position [45]. The ATR ranged from 0° to 30°, and the largest degree of trunk rotation was recorded.

The SRS-22 questionnaire is a widely used instrument to assess the health-related quality of life for patients with AIS [46], and the Chinese version was used in this study, which has been proven to be reliable and valid [47]. The SRS-22 questionnaire contained five domains: function, pain, self-image, mental health (five questions each), and satisfaction (two questions). Each question is scored from 1 to 5, where 1 is the worst, and 5 is the best, and the results are expressed as the mean score for each domain. Given the ceiling effect for the SRS-22 physical function, pain, and mental health domains [48], the self-image domain was used to assess self-image acceptability in AIS because of its ability to discriminate between subjects [49].

Apical vertebral rotation was assessed by the Nash-Moe method, which is a 5-scale grading system designed to describe the degree of regional vertebral rotation associated with scoliosis, where grade 0 represents the least rotation of the apical vertebra, and grade IV represents serious rotation [44].

Apical vertebral translation was measured as the distance (mm) by drawing a horizontal line through the center of the apical vertebra or disc centroid to the center sacral vertical line (CSVL).

The angle subtended by the upper border of the sacrum and the horizontal reference level indicated the degree of coronal pelvic obliquity [44].

### Statistical analysis

Sample size analysis was based on a two-sample independent t test using PASS 15 software. To determine the sample size for this study, estimates of the mean difference and standard deviation for the primary outcome of the concave/convex ratio were obtained from a pilot study of 12 patients who participated in the same program between June 2021 and November 2021. The mean difference and standard deviation were estimated to be 3% and 3.2%, respectively, in a two-tailed t test with an alpha level of 0.05 and a desired power of 80% to detect the difference between groups. Based on these assumptions and a dropout rate of 10%, we estimated a sample size of approximately 42 patients to be randomly assigned in a 1:1 ratio to the two groups.

Data were analyzed with SPSS 22.0. Analyses were performed in the intention-to-treat population, which included all the patients who were randomized regardless of having to take the treatment or not, and missing data were imputed using the last-observation-carried-forward (LOCF) method. The Shapiro Wilk test was performed to confirm the normal distribution of variables. Estimates of the treatment effect on each outcome were reported as the mean between-group difference with a 95% confidence interval (CI). The primary outcome was assessed by an analysis of covariance (ANCOVA), with the baseline value as the covariate. Secondary outcomes were analyzed with ANCOVA and the Chi-square test, as appropriate. Pearson and point-biserial correlations between the primary outcome and other secondary outcome measures were evaluated. We assessed the intraobserver variability of the radiological and anthropometric measurements using the intraclass correlation coefficient (ICC). All effects were estimated with a 95% CI and all statistical tests were two-tailed with α level of 0.05.

## Results

### Baseline characteristics and follow-up

From February 2022 through November 2022, a total of 68 patients were screened, of whom 26 patients who were excluded due to not meeting inclusion criteria or declined to participate (Fig 1) and 42 patients underwent randomization. Patients were randomly assigned to receive Schroth training (21 patients), or combined training (21 patients). The last patient completed the 24-week treatment period and was evaluated on April 7, 2023.

Three patients were lost to follow-up due to non-compliance with study protocol, and one patient was unable to appear at examination date due to logistic problems. A total of 10% (4/42) of cases had missing primary and secondary outcome variables at 24-week follow-up, and missing data were imputed using LOCF. The exercise training in both groups was well tolerated and there were no adverse events.

The mean age of the 42 enrolled adolescents was 13.33 (±2.41) years, and body mass index was 18.56 (±2.46) kg/m2. The age, sex, body mass index distribution, and baseline clinical characteristics did not differ between the two groups (Table 1). The intraobserver agreement was 0.98 for the baseline concave/convex ratio measurement, 0.93 to 0.99 for other baseline radiographic measurements, and 0.88 for the baseline ATR measurement; at 24 weeks, the intraobserver agreement was 0.96, 0.96 to 0.99, and 0.94 for each measurement, respectively.

**Table 1:** Demographic and baseline characteristics by randomized group.

Characteristic	Schroth (N = 21)	Combine(N = 21)
Age (years)	13.57±2.60	13.10±2.23
Female sex—no. (%)	17 (81)	15 (71.4)
Body-mass index (kg/m^2^)	18.70±2.24	18.42±2.72
Type of scoliosis—no. (%)		
Thoracic	4 (19)	4 (19)
Lumbar	10 (47.6)	7 (33.3)
Thoracolumbar	4 (19)	7 (33.3)
S-shaped	3 (14.3)	3 (14.3)
Risser grade—no. (%)		
0	7 (33.3)	5 (23.8)
1	1 (4.8)	0 (0)
2	0 (0)	5 (23.8)
3	4 (19.0)	3 (14.3)
4	7 (33.3)	6 (28.6)
5	2 (9.5)	2 (9.5)
Cobb angle (°)	17.67±4.02	17.82±4.81
Concave/convex ratio (%)	92.83±3.15	91.99±3.62
Pelvic obliquity (°)	1.58±0.86	1.60±0.93
Apical vertebral rotation—no. (%)		
Neutral (0)	6 (28.6)	4 (19.0)
Grade I	12 (57.1)	12 (57.1)
Grade II	3 (14.3)	5 (23.8)
Apical vertebral translation (mm)	14.01±7.61	17.21±9.71
Angle of trunk rotation (°)	6.19±2.04	6.26±2.37
SRS-22 Self-image (0–5 points)	3.81±0.73	3.83±0.66

The continuous data were presented in mean ± standard deviation (SD), number (n) and percentage (%) for categorical data.

### Primary outcome

For the combined training group, an increase at week 24 in the mean changes in concave/convex ratio from baseline was 3.91±0.57% (*P*<0.001), as compared with an increase of 0.45±0.76% (*P* = 0.56) in the Schroth training group. The mean difference between the combined training group and the Schroth training group at week 24 was 2.89% (95% CI, 1.58 to 4.20; *P*<0.001), as shown in Table 2.

**Table 2:** Primary and secondary efficacy outcome measures.

Outcome Measures	Baseline		Week 24		Mean within-group change ± SE		Between-group difference in mean change (95% CI)	
	Schroth	Combine	Schroth	Combine	Schroth	Combine	Combine minus Schroth	*p* value
	(n = 21)	(n = 21)	(n = 21)	(n = 21)	(n = 21)	(n = 21)		
**Primary Outcome**								
Concave/convex ratio (%)	92.83±3.15	91.99±3.62	93.27±2.09	95.90±2.54	0.45±0.76	3.91±0.57	2.89 (1.58 to 4.20)	<0.001
**Secondary Outcomes**								
Cobb angle (°)	17.67±4.02	17.82±4.81	15.48±4.42	14.00±5.87	-2.19±0.86	-3.81±0.62	-1.60 (-7.75 to 0.54)	0.14
Angle of trunk rotation (°)	6.19±2.04	6.26±2.37	4.71±1.98	3.50±2.27	-1.48±0.29	-2.76±0.42	-1.26 (-2.20 to -0.32)	0.01
SRS-22 Self-image (0–5 points)	3.81±0.73	3.83±0.66	3.97±0.53	4.13±0.52	0.16±0.07	0.30±0.07	0.149 (0.001 to 0.297)	0.049
Apical vertebral translation (mm)	14.01±7.61	17.21±9.71	11.12±5.36	12.32±8.34	-2.89±1.39	-4.89±1.38	-0.58 (-3.83 to 2.67)	0.72
Pelvic obliquity (°)	1.58±0.86	1.60±0.93	1.35±0.71	1.46±0.73	-0.23±0.12	-0.14±0.13	0.10 (-0.21 to 0.41)	0.52

Efficacy outcome measures were analyzed statistically using an ANCOVA model with baseline value as the covariate.

Data of both groups at baseline and at week 24 are presented as mean ± standard deviation.

SE, standard error; 95%CI = 95% confidence interval; ANCOVA, analysis of covariance.

### Secondary outcomes

As shown in Table 2, the mean change from baseline in Cobb angle at week 24 was a decrease of 3.81±0.62° (*P*<0.001) in the experimental group, as compared with a decrease of 2.19±0.86° (*P* = 0.02) in the control group. The mean difference between the two groups at week 24 was −1.60° (−7.75 to 0.54; *P* = 0.14).

At week 24, the mean change from baseline in the angle of trunk rotation was a decrease of 2.76±0.42° (*P*<0.001) in the experimental group, as compared with a decrease of 1.48±0.29° (*P*<0.001) in the control group. The mean difference between groups at week 24 was −1.26° (−2.20 to −0.32; *P =* 0.01).

The mean change from baseline in SRS-22 self-image at week 24 was an increase of 0.30±0.07 (*P*<0.001) in the experimental group, as compared with an increase of 0.16±0.07 (*P* = 0.03) in the control group. The mean difference between groups at week 24 was 0.149 (0.001 to 0.297; *P* = 0.049).

The mean changes from baseline in apical vertebral translation and pelvic obliquity at week 24 were a decrease of 4.89±1.38mm (*P* = 0.002) and 0.14±0.14° (*P* = 0.32) respectively in the experimental group, as compared with a decrease of 2.89±1.40mm (*P* = 0.052) and 0.23±0.13° (*P* = 0.09) respectively in the control group. The mean change in apical vertebral translation was −0.58mm (−3.83 to 2.67; *P* = 0.72) and in pelvic obliquity was 0.10° (−0.21 to 0.41; *P* = 0.52) between the experimental group and the control group, which did not differ significantly.

As shown in Table 3, the apical vertebral rotation improved from baseline by at least one class in 3 patients (14.3%) in the control group, and in 9 patients (42.9%) in the combined training group, and combined training was estimated to be more favorable than Schroth training (*P* = 0.04).

**Table 3:** The between-group difference for change in apical vertebral rotation.

Secondary Outcome	Schroth	Combine	*p* value
	(N = 21)	(N = 21)	
Apical vertebral rotation—no. (%)			0.04
Patients with improvement of at least one class	3 (14.3)	9 (42.9)	

The secondary outcome measure was analyzed with the Chi-square test.

#### Relationship between changes in the concave/convex ratio and other secondary outcome measures

Highly statistically significant correlations were found between the primary outcome measure change in concave/convex ratio and two secondary outcome measures including change in angle of trunk rotation and improvement in apical vertebral rotation (*r* = 0.605, *r* = 0.566, respectively; *P*<0.001). No significant correlations were found between the change in concave/convex ratio and Cobb angle (*r* = 0.109; *P* = 0.49), apical vertebral translation (*r* = 0.106; *P* = 0.50), pelvic obliquity (*r* = -0.045; *P* = 0.78) or SRS-22 self-image (*r* = 0.173; *P* = 0.27).

## Discussion

In this trial, 24 weeks of Schroth exercises in conjunction with an intervention program for pelvic rotation correction resulted in an improvement in the concave/convex ratio that was significantly greater than that of Schroth exercises alone in adolescents with mild idiopathic scoliosis. A greater degree of improvement in both trunk rotation angle and apical vertebral rotation was also observed compared to Schroth exercises alone.

The pelvis has been described as the "pelvic vertebra" in that it is intimately intertwined with the alignment of the spine. Several studies have revealed a relationship between pelvic asymmetry and spinal deformity in patients with AIS and have suggested that static pelvic axial rotation is correlated with apex vertebral rotation [16, 24]. Consistent with previous studies, the present study found that the primary outcome measure change in the concave/convex ratio and improvements in the angle of trunk rotation and apical vertebral rotation were highly associated, and the improvements in all secondary measures support the clinical significance of the observed improvements in pelvic rotation. Such a convergence of evidence suggests that the inclusion of the pelvis in the analysis of the spine is essential and that it may be beneficial to include pelvic rotation treatment in the management of patients with AIS.

The pelvis has not only served as a static link between the lower extremities and the trunk, but has also contributed to the overall dynamic postural stability from a biomechanical standpoint, an initial malalignment of the pelvis in the axial plane would affect the overall dynamics of the trunk-pelvis kinematic chain during movement [26]. Pasha S et al. [26] investigated pelvic orientation during different trunk movements in adolescents with mild right thoracic and right thoracic-left lumbar scoliosis. The authors found that a majority of the patients in the 2 scoliotic groups had the pelvis rotated to the side of the major curve (right), as a result, during movements in the direction opposite to the major curve (axial rotation movement to the left), they used more pelvic rotation to the left to compensate for the initial rotation of the pelvis in the opposite direction. Besides, some authors have found that even in mild scoliosis curves (Cobb angle <20°) [27], patients with AIS have asymmetrical gait patterns in the axial plane compared to normal controls [27–31], with a lesser proportion of the shoulder line turning to the left and a contra-rotation of the pelvis to the right in adolescents with right thoracic idiopathic scoliosis [30]. On the other hand, as pain is an important issue in patients with AIS [50, 51], pelvic rotational asymmetry may predispose patients with AIS to low back pain [32]. Studies have shown that pelvic rotational asymmetry would result in asymmetric axial rotation of the lumbar vertebrae, which is associated with low back pain due to decreased postural stability [33, 52, 53].

Given the importance of initial pelvic alignment in the transverse plane in overall static and dynamic postural stability, management of pelvic axial rotation may be the first step to anticipate appropriate and individualized treatment of AIS; incorporating pelvic rotation correction into regular physical therapy for spinal deformity may help to design a more effective treatment plan. However, to our knowledge, the present work is the first randomized controlled trial to evaluate the effect of PNF-based physical therapy on pelvic rotation and spinal deformities in patients with mild AIS. A previous randomized controlled trial by Abdel-Aziem et al. [54] included hippotherapy in the treatment of adolescents with idiopathic scoliosis. They showed that hippotherapy combined with Schroth exercises was more effective than Schroth exercises alone in improving the scoliotic angle, pelvic torsion, and vertical spinal rotation using raster stereographic assessment. The author suggested that horseback riding increases spinal alignment by creating a rotational pelvic motion along the longitudinal axis. Consistent with this study, the results of our study showed that the integration of PNF-based pelvic rotation correction into Schroth exercises helps to achieve more noticeable improvements in pelvic axial rotation and spinal alignment. In addition, compared with the relatively expensive hippotherapy approach, PNF-based physical therapy may be more accessible to adolescents with idiopathic scoliosis in clinical practice.

Possible mechanisms underlying the treatment of asymmetry in pelvic axial rotation in patients with AIS may be attributed to the correction of the trunk and pelvic-femoral muscle imbalance. Mahaudens P et al. [27] reported asymmetric electromyographic activities of the quadratus lumborum, erector spinae, gluteus medius, rectus femoris, semitendinosus, tibialis anterior, and gastrocnemius muscles in both static and dynamic electromyographic measurements in AIS, reflecting an asymmetric contraction of these muscles. This muscular asymmetry may be explained as a compensatory response to maintain the static and dynamic equilibrium of the spine and pelvis [29, 55]. Similarly, Begon et al. [56] proposed that pelvic asymmetries may alter the line of action of the muscles connecting the pelvis to the spine and were associated with the trunk muscle imbalance in AIS. In addition, Doran M et al. [57] evaluated the symmetry of abdominal muscle thickness in AIS using ultrasonography, he suggested that the asymmetries in the abdominal muscles might be related to the rotation of pelvic asymmetries, where all abdominal muscles were linked with the pelvic bones (in terms of origin or insertion) [58]. Therefore, it would be expected that conservative management by modifying the muscle activation strategy could correct the imbalance of these paravertebral and pelvic-femoral muscles [27, 59] and improve the initial pelvic alignment in the transverse plane in adolescents with scoliosis. As a conservative treatment, the PNF technique may be an appropriate option to correct the paravertebral and pelvic-femoral muscle imbalance for AIS. PNF stretching involves a theory of reciprocal inhibition that is used to fully stretch the relatively tight muscle groups and activate the relatively weak muscle groups [35, 37], and a pelvic pattern of PNF treatment could theoretically provide symmetrical activation of the muscles connecting the pelvis to the spine and improve the spatial alignment of the pelvis and trunk in patients with AIS.

Few studies incorporating the PNF technique in the treatment of pelvic rotation in patients with AIS have been published. A case-series study by Stępień et al. [38] showed that the PNF technique improved the symmetry of mobility in the transverse plane in the trunk-pelvis-hip complex and reduced the angle of trunk rotation in adolescent girls with idiopathic scoliosis. This evidence suggests that the PNF mobilization technique has a direct effect on pelvic axial symmetry; however, the strength of the findings is limited by the lack of a control group and radiographic evidence to confirm the efficacy of the interventions applied. Similarly, the present randomized controlled trial showed significant improvement in the concave/convex ratio, apical vertebral rotation, and trunk rotation angle, indicating that the use of PNF in pelvic rotation treatment for patients with AIS is justified and beneficial. Furthermore, although the Schroth treatment program includes pelvic correction by rotating the pelvis horizontally to correct excessive rotation, unlike the PNF stretching technique, pelvic correction in the Schroth treatment served only as a starting point for active contraction of the trunk muscles [60], and its importance in the treatment process was not emphasized as much, which may explain why no improvements from baseline were seen in the concave/convex ratio in the Schroth training group.

Qiu Y et al. [24] showed that the pelvic concave/convex ratio was also correlated with the lumbar Cobb angle; however, the present study did not find any association between the concave/convex ratio and the main Cobb angle. The Cobb angle of participants in both groups remained stable at the end of treatment, consistent with previous literature findings showing average declines of 2~3° in Cobb degrees after 24 weeks of Schroth training in mild and moderate AIS [10, 61]. Possible reasons could be the small curves of the AIS participants in the present study and the lack of long-term follow-up. While the long-term treatment effect on the Cobb angle is important to address in future studies, deformity parameters such as pelvic axial rotation should not be ignored either, as they are considered to accurately reflect the treatment efficacy and clinical status of the patient [22, 23], and are essential in the management of adolescent idiopathic scoliosis, especially in patients with mild scoliosis who have a lower risk of curve progression [2, 62].

The goals of treatment for idiopathic scoliosis are not only to prevent curve progression and achieve curve correction but also to restore a healthy self-image, a key component of health-related quality of life [63–66]. Schreiber et al. [67] claimed that supervised Schroth exercises were associated with improved self-image when compared with treatment controls. Similar results were found in the present study, both groups with mild scoliotic AIS showed a significant improvement from baseline in their self-image scores. The Schroth exercise protocol used in both groups in the current study helped to improve their physical status (i.e., strength and elasticity of certain muscles) [4, 68, 69]; improvements in physical function and vitality may contribute to significantly improved self-image scores in both groups. We assumed that patients in the experimental group would be more confident about their body image due to the longer training time and more comprehensive treatment, however, the lower limit of the confidence interval for the between-group difference in self-image was small, which may be due to the short duration of treatment and the insufficient sample size. Another possible reason may be that the questionnaire is not specific enough to address the perception of whether the improvement of the spine’s appearance was in rib prominence, trunk shift, shoulder asymmetry, or some other area such as pelvic asymmetry [70]. Some studies have recommended the Spinal Appearance Questionnaire as a valid instrument to assess the self-image in patients with AIS [71, 72], as it provides more detail than the SRS in the appearance domain, and provides explanation of spinal deformity’s concerns and improvements [70].

Moreover, pelvic obliquity is frequently observed in patients with AIS [73–75], and Banno T et al. [75] claimed that postoperative coronal decompensation occurred more frequently in patients with preoperative pelvic obliquity than in those without preoperative pelvic obliquity. The present study also explored the effect of the corrective PNF program on pelvic obliquity, but no significant differences were found between groups or within groups. Doran M et al. [57] claimed that asymmetries of the abdominal muscles could be related to asymmetries of the pelvis, such as rotation or tilt. However, abdominal muscle asymmetries were not adequately addressed in the present study because the applied PNF technique was primarily designed to target specific muscles involved in pelvic axial rotation, such as hip adductors, hip abductors, and quadratus lumborum, which may explain the lack of effective reduction of pelvic obliquity.

This trial has some limitations. First, the duration of the trial was brief at 24 weeks, and the long-term effects of pelvic rotation correction on curve progression would still be important to address in future trials. Despite this limitation, the present study indicated that the correction of pelvic rotation had an indispensable role in the management of three-dimensional spinal and trunk deformities in patients with mild AIS. Second, the evaluation of PAR on posteroanterior radiographs would be influenced by the patient’s position. The method of calculating the concave/convex ratio has been documented in previous studies [16, 18, 19, 21, 24], and the intra-observer agreement was also good; therefore, the measurement of pelvic axial rotation on posterior-anterior radiographs would not be a perfect method but a practical method to assess the pelvic position [18]. Third, we only assessed the spinal deformities in the axial and coronal planes. Given the complicated effects of pelvic rotation on spinal alignment in three-dimensional space, it would be difficult to analyze the change in the body in all three planes; therefore, the effects in sagittal planes and their associations with clinical outcomes would be worth exploring in further studies. Finally, since no significant difference was found between the two groups on self-image scores, the incremental benefit of combined training is not large enough to warrant recommending it over Schroth training when aiming to improve self-image scores. Future studies with longer follow-up and larger sample sizes are needed to validate the effect of the combined treatment on body image, on the other hand, a more detailed instrument, such as the Spinal Appearance Questionnaire, is needed to respond to changes in the patient’s spinal appearance.

## Conclusions

The PNF-based pelvic rotation correction exercise program combined with the Schroth exercise improved pelvic axial rotation more than Schroth alone. The benefits of the combined training were also evident in the improvement of trunk and spinal deformities, including trunk rotation angle and apical vertebral rotation. Pelvic rotation correction may be an effective treatment option for improving pelvic and spinal deformities in mild AIS, and further studies are needed to investigate its long-term effects.

## Supporting information

S1 Checklist CONSORT checklist. (DOCX)

S1 File Schroth training program. I. Schroth-specific exercises, II. Postural re-education incorporated into activities of daily livings.
(PDF)

S2 File Training procedure of corrective exercise program for pelvic rotation. (PDF)

S3 File Study protocol (Original Chinese version). (DOCX)

S4 File Study protocol (English version). (DOCX)

S1 Data Raw data. (XLSX)
