# Supplementary material for: Pelvic rotation correction combined with Schroth exercises for pelvic and spinal deformities in mild adolescent idiopathic scoliosis: A randomized controlled trial
Source: PLoS One. 2024 Jul 30;19(7):e0307955. doi: 10.1371/journal.pone.0307955 (PMC11288462; doi:10.1371/journal.pone.0307955)
Supplement: S1 File — I. Schroth-specific exercises, II. Postural re-education incorporated into activities of daily livings. (PDF) [file pone.0307955.s002.pdf]

S1. Schroth training program

I. Schroth-specific exercises

The 50x exercise	The frog at the pond
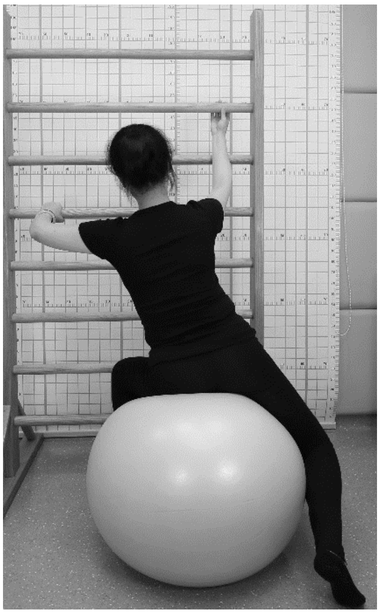	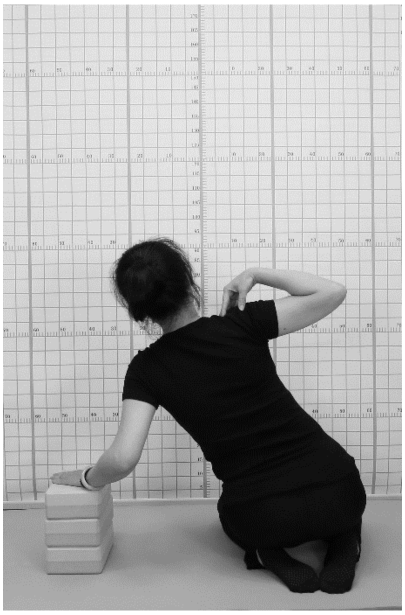
Muscle Cylinder	The door handle exercise
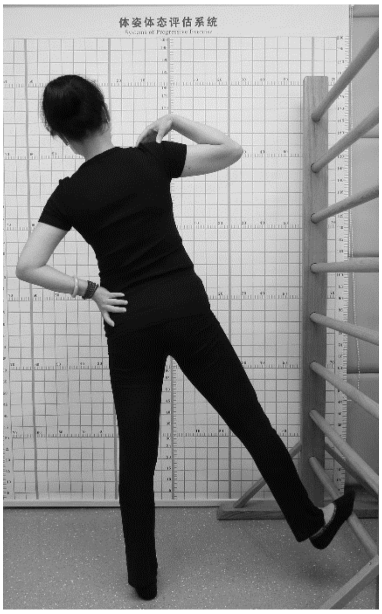	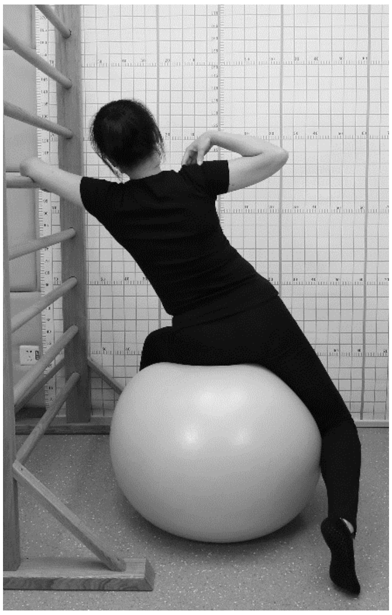

II. Postural re-education incorporated into activities of daily livings

sitting position	standing position	standing while leaning against something
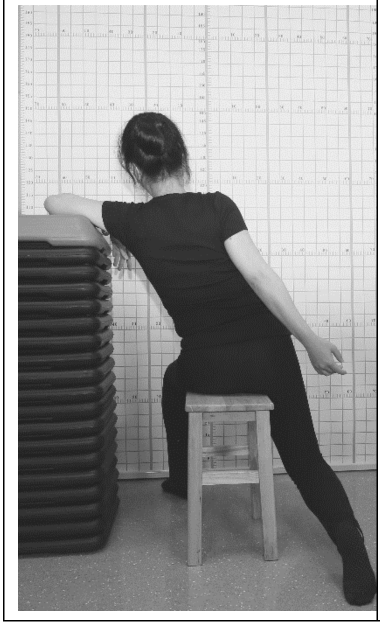	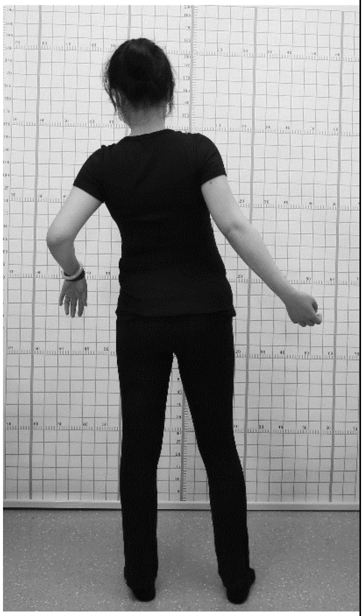	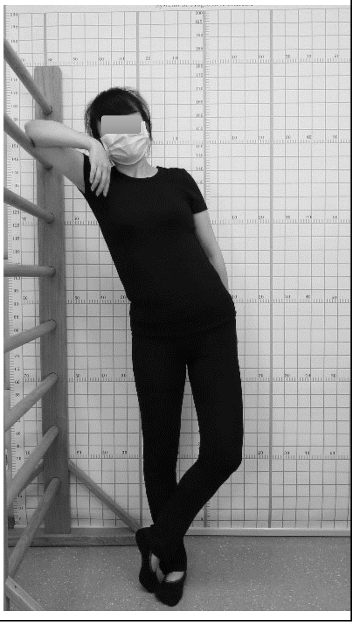

Note: Exercises shown above use the right thoracic-left lumbar scoliosis as an example.
