# Supplementary material for: Pelvic rotation correction combined with Schroth exercises for pelvic and spinal deformities in mild adolescent idiopathic scoliosis: A randomized controlled trial
Source: PLoS One. 2024 Jul 30;19(7):e0307955. doi: 10.1371/journal.pone.0307955 (PMC11288462; doi:10.1371/journal.pone.0307955)
Supplement: S2 File — (PDF) [file pone.0307955.s003.pdf]

S2. Training procedure of corrective exercise program for pelvic rotation

**I. Stretch of asymmetric muscles of the pelvis**	
**a. Sagittal plane**	
Hip flexors (psoas, rectus femoris muscles)	
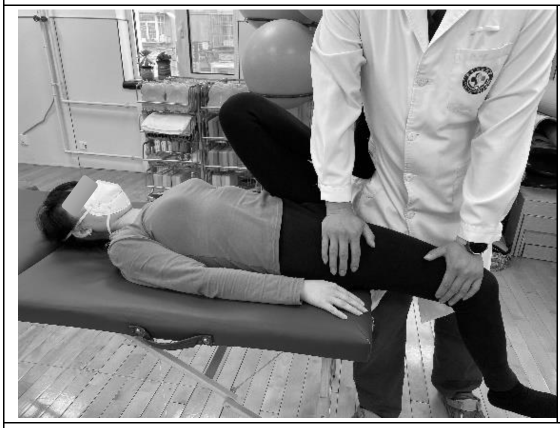	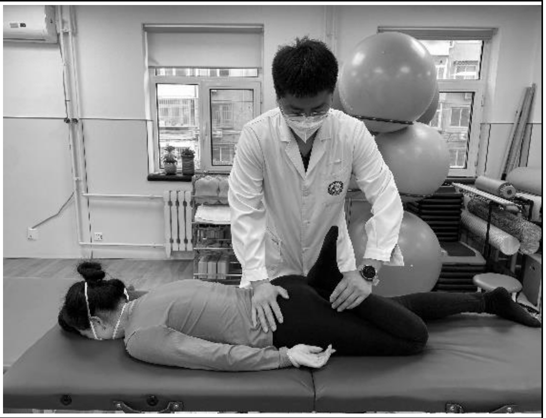
Hip extenders (hamstrings, gluteus maximus muscles)	
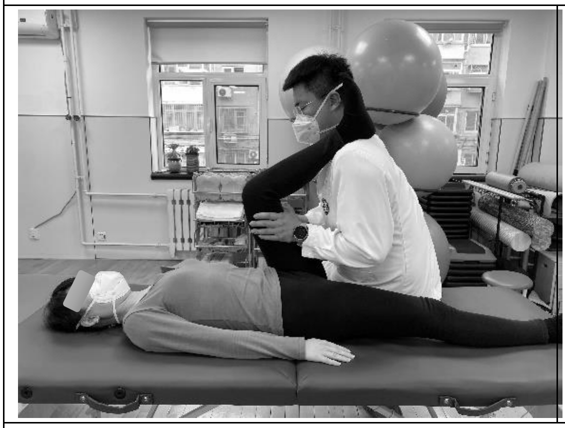	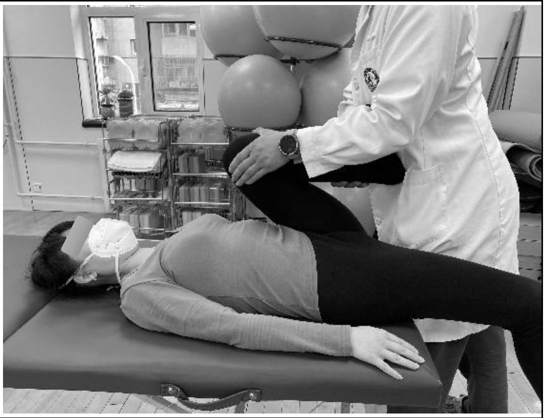
**b. Coronal plane**	
Hip adductors	Hip abductors (tensor fascia lata, gluteus medius muscles)
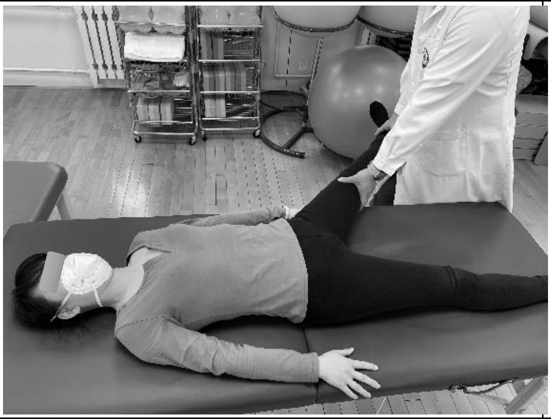	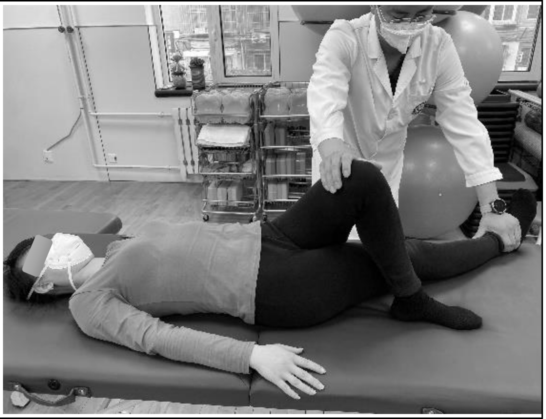
Quadratus lumborum	

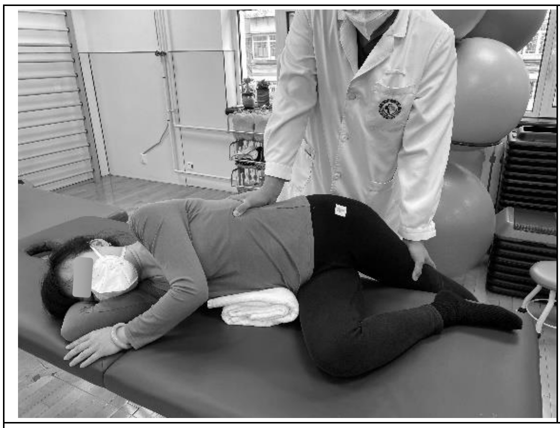	
**c. Transverse plane**	
Lateral rotators (piriformis, superior and inferior gemellus, obturator internus, and quadratus femoris muscles)	Medial rotators (medial hamstrings)
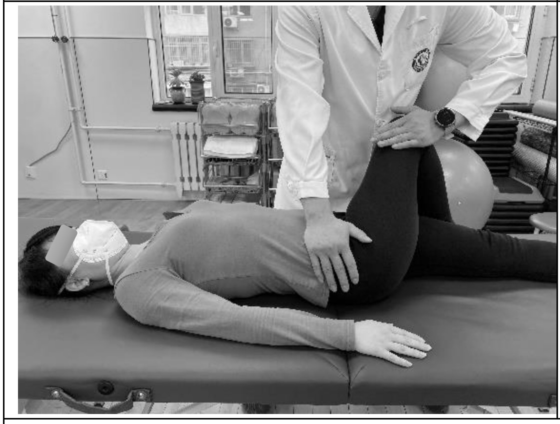	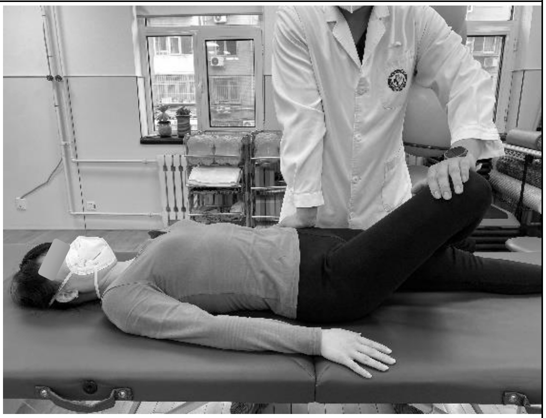
**II. De-rotation stretching of the pelvic girdle**	
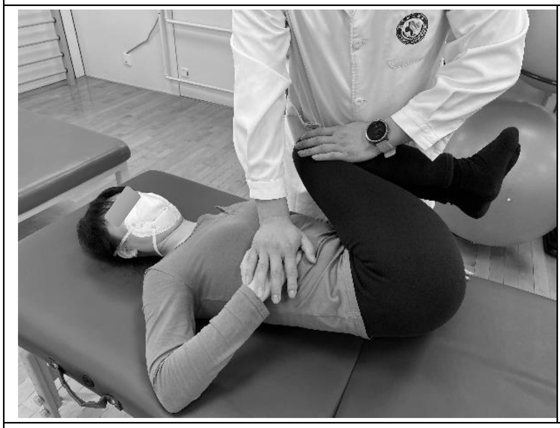	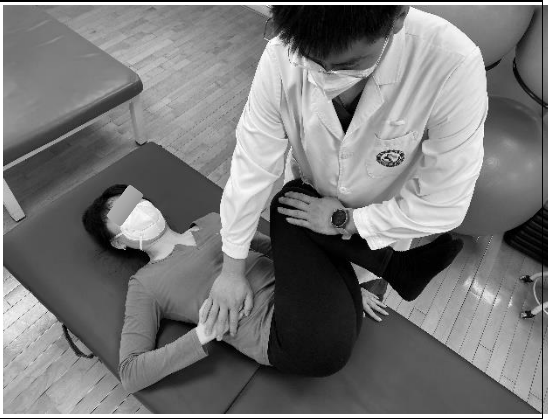
**III. Motor control exercises**	

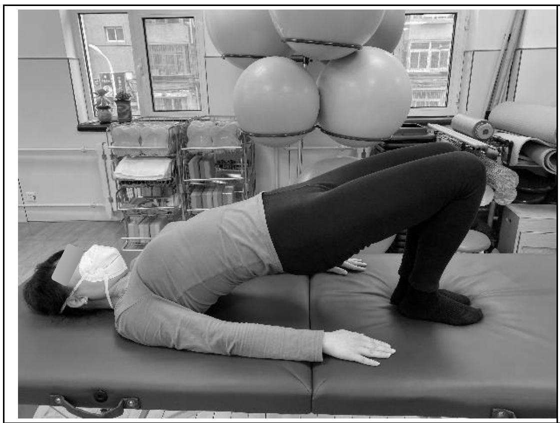	
Front view	Side view
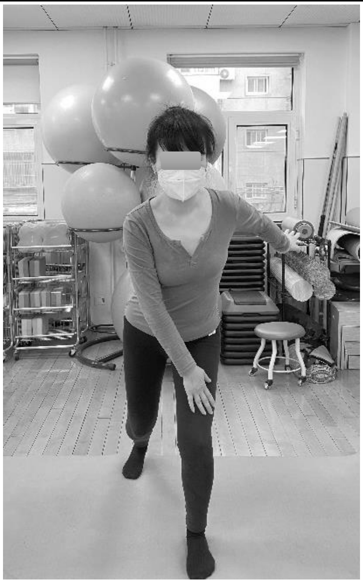	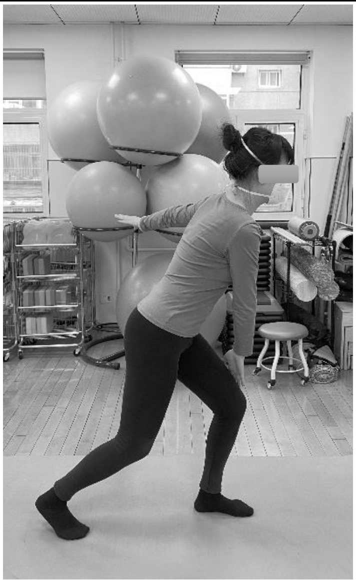
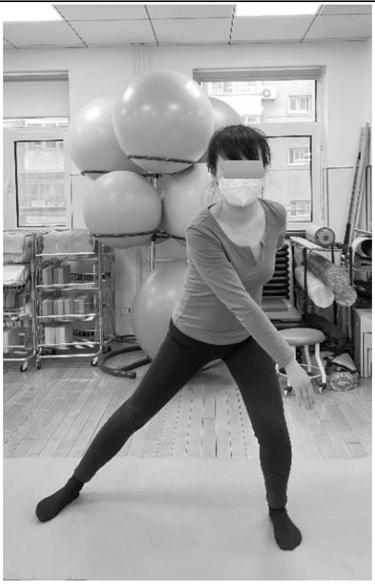	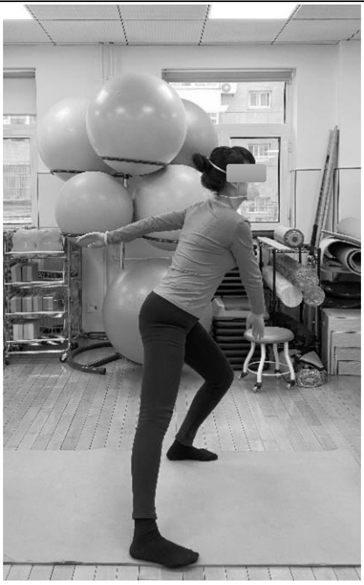

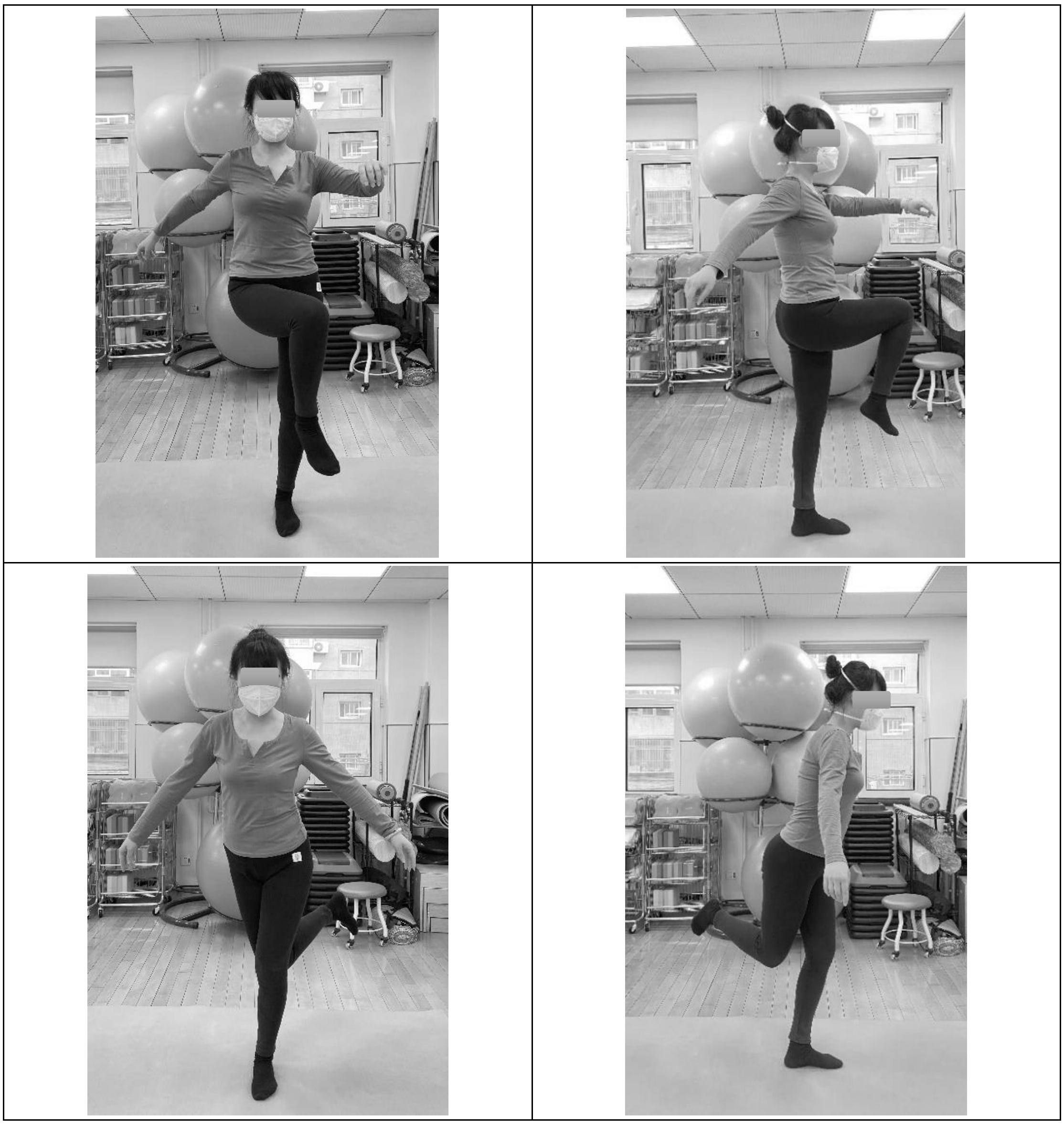

Note: All muscles of pelvis shown above are assessed with a sequence of physical tests prior to the stretch therapy, where only the short and tight muscles are identified and treated, while motor control exercises are to be carried out bilaterally.
