# Supplementary material for: Pelvic rotation correction combined with Schroth exercises for pelvic and spinal deformities in mild adolescent idiopathic scoliosis: A randomized controlled trial
Source: PLoS One. 2024 Jul 30;19(7):e0307955. doi: 10.1371/journal.pone.0307955 (PMC11288462; doi:10.1371/journal.pone.0307955)
Supplement: S3 File — (DOCX) [file pone.0307955.s004.docx]

| **项 目 编 号** |
| --- |
| HP2022-50-507005 |

**北京市海淀区卫生健康发展科研培育计划**

**任务书**

项目名称：“3+1”多维运动疗法对轻度青少年特发性脊柱侧凸曲线进展影响的研究

专业类别：中医（ ）西医（√）管理（ ）

转化项目: 是( ) 否(√)

申请单位：航天中心医院

申 请 人：张亚菲

联 系 人：张亚菲

联系电话：13436639195

起止年月： 2022 年 1 月至 2023 年 12月

**一、项目基本信息**

| **项**  **目**  **信**  **息** | 项目名称 | “3+1”多维运动疗法对轻度青少年特发性脊柱侧凸曲线进展影响的研究 |
| --- | --- | --- |
|  | 关键词（≤5个） | 青少年特发性脊柱侧凸，骨盆，轴向旋转，运动治疗 |
|  | 项目类型 | □基础研究 🗹临床研究 |
| **项目摘要（限400字）：** | | |
| 青少年特发性脊柱侧弯是青少年最常见的脊柱三维结构畸形，特异性运动疗法可有效减少冠状面脊柱侧弯的角度，但在躯干轴向畸形方面的效果并无足够的证据支持。有研究表明，骨盆的轴向旋转可能与特发性脊柱侧弯的进展有关，并且，骨盆旋转的纠正对躯干的三维结构畸形的改善有一定的治疗作用。但目前缺少通过矫正骨盆轴向旋转来治疗脊柱侧弯三维畸形的高质量临床研究。因此，本研究拟通过一项单中心、随机对照试验设计，对轻度青少年脊柱侧凸患者随机进行“3+1”多维运动治疗（Schroth治疗联合骨盆旋转矫正）或仅进行Schroth治疗，评估治疗后24周“3+1”多维运动治疗组与Schroth治疗组在改善骨盆旋转参数、Cobb角度、躯干旋转角度等骨盆和脊柱三维结构参数方面的差异，以及在SRS-22问卷中自我形象认知等方面改善情况的差异，验证“3+1”多维运动疗法在改善轻度青少年特发性脊柱侧凸的躯干三维平衡和日常生活质量方面的疗效。本研究将为青少年特发性脊柱侧凸的治疗策略提供临床证据。 | | |

**二、立题依据**

| **1、研究目的与意义**  青少年特发性脊柱侧凸（adolescent idiopathic scoliosis，AIS）是发生在生长发育阶段健康儿童或青少年的脊柱和躯干的三维结构畸形，通常会出现脊柱侧凸并伴有脊柱轴位扭曲及矢状面曲线的改变[1, 2]。国际脊柱侧弯研究协会（Scoliosis Research Society , SRS）将站立位脊柱正位X线片中测量的柯布角度（Cobb）≥10°作为脊柱侧凸的诊断标准[3]，其在全球、亚洲地区及国内部分地区的患病率统计数据均显示为2%左右[4]。AIS常于青春前期或骨骼成熟前发生，通常女孩在10-14岁、男孩12-16岁是发病的高风险期，处于生长发育高峰期的骨骼未发育成熟的儿童和青少年具有极高的进展风险[5-7]。脊柱畸形的快速发展不仅导致生长过程中形体外观改变，还会对青少年心理健康和生活质量造成负面影响，大角度侧弯还会导致肺部并发症和疼痛，最终造成成年后健康障碍及手术的风险增加[8-10]。  AIS的病因及发病机制尚不明确。骨盆可能在脊柱侧弯的发病及进展中起到关键作用[11-14]。Jeff L. Gum [15] 认为骨盆的轴位旋转与胸椎侧凸有关，在伴有胸腰段或腰段代偿弧的AIS患者中，骨盆轴位旋转方向和主胸弯方向相同来维持脊柱骨盆的整体平衡。Xu-Sheng Qiu [16] 等人证实AIS患者脊柱旋转伴随着骨盆轴向旋转，表现为髋骨宽度的凹/凸比不等。  骨盆作为所有的腹肌及髂肌的起止点，在青春期发育迅速时，腹肌的结构和力量失衡与骨盆的失衡互为干扰[17]。已有研究[17-19]指出AIS患者的腹部相对肌肉厚度比健康青少年更不对称，这种不对称可能与骨盆旋转相关，并可能是脊柱侧弯纵轴旋转的原因之一，因此，骨盆旋转的干预有可能影响到包括腹肌在内的周围附着肌群的结构。另一方面，骨盆的不对称在脊柱侧弯的进展中起到关键作用，作为连接脊柱和下肢的关键结构，骨盆对称性对于脊柱侧凸骨性结构对称性及下肢平衡能力有重要的临床意义[20]。  **2、国内外研究现状分析及存在问题**  AIS保守治疗的主要目标是阻止和延缓Cobb角进展，此外，纠正与脊柱侧凸相关的三维躯干畸形也是保守治疗的一个重要问题。Cobb角被视为评估脊柱侧弯畸形的金标准，而对于轻度AIS患者来说，Cobb角既不是独特的也不是唯一的决定性参数。近年来，以德国施罗斯（Schroth）为代表的脊柱侧凸特异性运动疗法在轻中度AIS患者中的疗效已得到众多的高等级临床证据支持，通过脊柱侧凸特异性物理治疗，轻中度脊柱侧凸患者的冠状面畸形已经得到了很好的控制[21-23]，但在躯干轴向畸形方面的效果并无足够的证据支持。  骨盆轴向旋转可能与胸椎或胸腰椎的椎体旋转等脊柱畸形有关[15, 24, 25]，Begon等人认为骨盆的不对称性与脊柱侧弯的进展相关[26]，邱勇等人也提出术前存在骨盆轴向旋转的AIS患者，术后发生冠状面失衡的风险更大[27]。并且，目前越来越多的研究开始关注躯干三维畸形的治疗，有研究表明骨盆旋转的纠正对躯干的三维结构畸形的改善有一定的治疗作用。  在一项随机对照试验中，Abdel-azie等人证明了为期10周的马术训练和Schroth运动结合治疗改善了AIS患者的姿势不对称性，如脊柱侧弯角度、骨盆轴向旋转和椎体轴向旋转[20]。作者认为马术训练对脊柱三维畸形改善的的潜在机制可能是马在行走过程中产生了与人类骨盆相似的三维运动，马的运动使骑在上面的侧弯患者的骨盆产生沿着纵轴的旋转运动，继而向上产生脊柱的弯曲和旋转。然而，马术训练是一种相对昂贵的方法，可能对大部分中国的青少年来说并不适用。  本体感受性神经肌肉促进（PNF）拉伸技术常用于患有包括AIS骨盆轴向不对称在内的运动问题的患者中，以增强由连接骨盆和脊柱的不平衡肌肉引起的旋转活动范围[28-31]。由Stypien等人进行的一项干预性研究表明，PNF拉伸对患有青少年特发性脊柱侧凸的女孩的躯干旋转角度有短时的改善，表明PNF拉伸可能是纠正AIS横向平面上躯干和骨盆不对称的有效方法[28]，但是因为该研究中缺乏影像学证据以及未设置对照组，降低了该研究结果在临床应用中的外推性。  **3、应用方向或应用前景**  前期本研究团队在本研究中心开展了“3+1”多维运动治疗青少年特发性脊柱侧凸的试点研究，即将骨盆旋转矫正纳入脊柱侧弯Schroth治疗中，联合矫正AIS患者的骨盆和躯干的三维结构畸形（图1）。我们对12名平均年龄13.6岁，Cobb角在10°～25°的青少年特发性脊柱侧凸患者进行了为期24周的治疗。治疗结束后评估发现在骨盆轴向对称性、躯干旋转角指标、椎体轴向旋转、Cobb角等方面，接受了Schroth治疗联合骨盆旋转矫正的AIS患者有更明显改善，这为我们的研究工作提供了有力的结果支撑。因此，本研究拟应用前瞻性随机对照研究设计，探索“3+1”多维运动疗法对治疗轻度青少年特发性脊柱侧凸的躯干三维结构畸形的有效性，为青少年特发性脊柱侧凸的治疗策略提供临床证据。  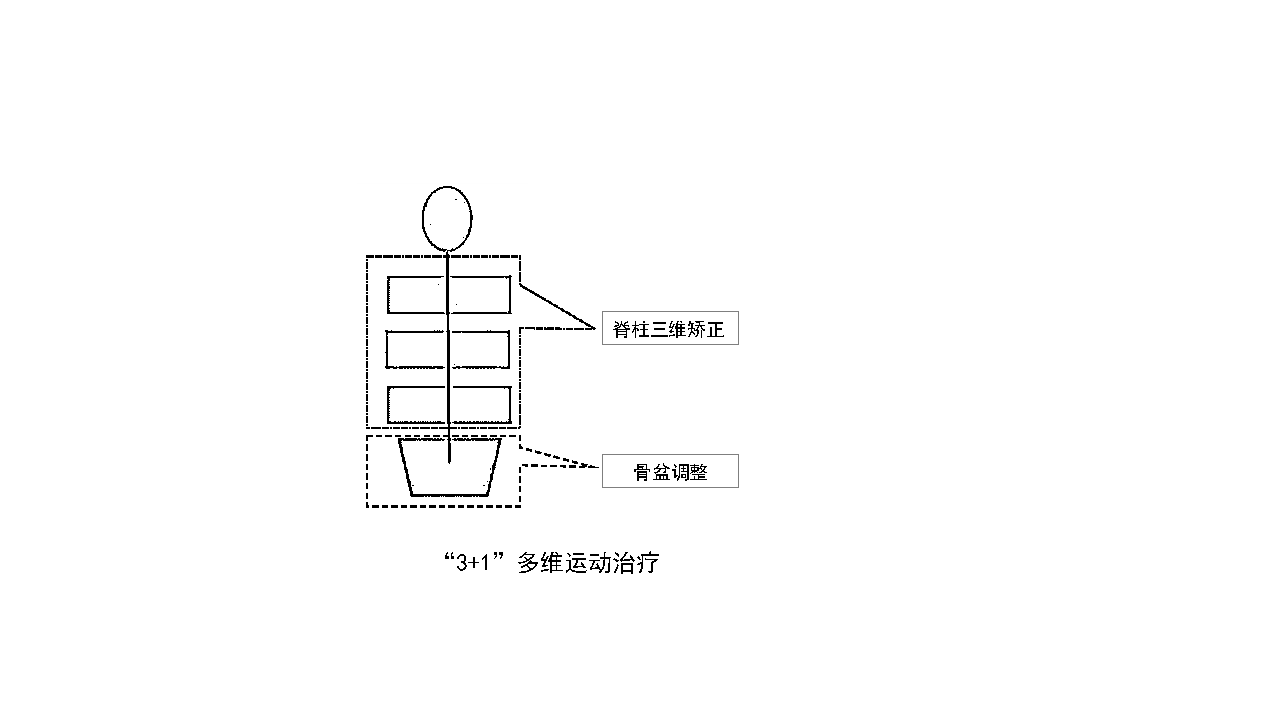  图1. “3+1”多维运动治疗技术示意图  注：“3”-脊柱三维矫正（Schroth治疗）；“1”-骨盆对称性矫正。  **参考文献**  **（参考文献格式：论文：作者，题目，刊名，年份，卷（期），起止页码；**  **专著：作者，书名，出版者，年份。）**  1. Negrini S, Donzelli S, Aulisa AG, Czaprowski D, Schreiber S, de Mauroy JC et al. 2016 SOSORT guidelines: orthopaedic and rehabilitation treatment of idiopathic scoliosis during growth. Scoliosis Spinal Disord. 2018;13:3.  2. Hresko MT. Clinical practice. Idiopathic scoliosis in adolescents. N Engl J Med. 2013;368(9):834-41.  3. Weinstein SL, Dolan LA, Cheng JC, Danielsson A, Morcuende JA. Adolescent idiopathic scoliosis. Lancet. 2008;371(9623):1527-37.  4. Zhang H, Guo C, Tang M, Liu S, Li J, Guo Q et al. Prevalence of scoliosis among primary and middle school students in Mainland China: a systematic review and meta-analysis. Spine (Phila Pa 1976). 2015;40(1):41-9.  5. Loncar-Dusek M, Pecina M, Prebeg Z. A longitudinal study of growth velocity and development of secondary gender characteristics versus onset of idiopathic scoliosis. Clin Orthop Relat Res. 1991(270):278-82.  6. Busscher I, Kingma I, de Bruin R, Wapstra FH, Verkerke GJ, Veldhuizen AG. Predicting the peak growth velocity in the individual child: validation of a new growth model. Eur Spine J. 2012;21(1):71-6.  7. Lonstein JE, Carlson JM. The prediction of curve progression in untreated idiopathic scoliosis during growth. J Bone Joint Surg Am. 1984;66(7):1061-71.  8. Di Felice F, Zaina F, Donzelli S, Negrini S. The Natural History of Idiopathic Scoliosis During Growth: A Meta-Analysis. Am J Phys Med Rehabil. 2018;97(5):346-56.  9. Koumbourlis AC. Scoliosis and the respiratory system. Paediatr Respir Rev. 2006;7(2):152-60.  10. Tones M, Moss N, Polly DW, Jr. A review of quality of life and psychosocial issues in scoliosis. Spine (Phila Pa 1976). 2006;31(26):3027-38.  11. Karski T. Etiology of the so-called "idiopathic scoliosis". Biomechanical explanation of spine deformity. Two groups of development of scoliosis. New rehabilitation treatment; possibility of prophylactics. Stud Health Technol Inform. 2002;91:37-46.  12. Fiala L. [Etiology of the so-called idiopathic scoliosis]. Bratisl Lek Listy. 1954;34(12):1452-6.  13. Saji MJ, Upadhyay SS, Leong JC. Increased femoral neck-shaft angles in adolescent idiopathic scoliosis. Spine (Phila Pa 1976). 1995;20(3):303-11.  14. Burwell RG, Cole AA, Cook TA, Grivas TB, Kiel AW, Moulton A et al. Pathogenesis of idiopathic scoliosis. The Nottingham concept. Acta Orthop Belg. 1992;58 Suppl 1:33-58.  15. Gum JL, Asher MA, Burton DC, Lai SM, Lambart LM. Transverse plane pelvic rotation in adolescent idiopathic scoliosis: primary or compensatory? Eur Spine J. 2007;16(10):1579-86.  16. Qiu XS, Zhang JJ, Yang SW, Lv F, Wang ZW, Chiew J et al. Anatomical study of the pelvis in patients with adolescent idiopathic scoliosis. J Anat. 2012;220(2):173-8.  17. Doran M, Öneş K, Terzibaşioğlu AM, Çinar Ç, Ata İ. Ultrasonographic evaluation of abdominal muscle thickness symmetry in adolescent idiopathic scoliosis: a case-controlled study. Eur J Phys Rehabil Med. 2021;57(6):968-76.  18. Linek P, Saulicz E, Kuszewski M, Wolny T. Ultrasound Assessment of the Abdominal Muscles at Rest and During the ASLR Test Among Adolescents With Scoliosis. Clin Spine Surg. 2017;30(4):181-6.  19. Ma CZ, Ren LJ, Cheng CL, Zheng YP. Mapping of Back Muscle Stiffness along Spine during Standing and Lying in Young Adults: A Pilot Study on Spinal Stiffness Quantification with Ultrasound Imaging. Sensors (Basel). 2020;20(24).  20. Abdel-Aziem AA, Abdelraouf OR, Ghally SA, Dahlawi HA, Radwan RE. A 10-Week Program of Combined Hippotherapy and Scroth's Exercises Improves Balance and Postural Asymmetries in Adolescence Idiopathic Scoliosis: A Randomized Controlled Study. Children (Basel). 2021;9(1).  21. Kuru T, Yeldan İ, Dereli EE, Özdinçler AR, Dikici F, Çolak İ. The efficacy of three-dimensional Schroth exercises in adolescent idiopathic scoliosis: a randomised controlled clinical trial. Clin Rehabil. 2016;30(2):181-90.  22. Liu D, Yang Y, Yu X, Yang J, Xuan X, Yang J et al. Effects of Specific Exercise Therapy on Adolescent Patients With Idiopathic Scoliosis: A Prospective Controlled Cohort Study. Spine (Phila Pa 1976). 2020;45(15):1039-46.  23. Negrini S, Donzelli S, Negrini A, Parzini S, Romano M, Zaina F. Specific exercises reduce the need for bracing in adolescents with idiopathic scoliosis: A practical clinical trial. Ann Phys Rehabil Med. 2019;62(2):69-76.  24. Zhao Y, Qi L, Yang J, Zhu X, Yang C, Li M. Factors affecting pelvic rotation in idiopathic scoliosis: Analysis of 85 cases in a single center. Medicine (Baltimore). 2016;95(46):e5458.  25. Wang ZW, Wang WJ, Sun MH, Liu Z, Zhu ZZ, Zhu F et al. Characteristics of the pelvic axial rotation in adolescent idiopathic scoliosis: a comparison between major thoracic curve and major thoracolumbar/lumbar curve. Spine J. 2014;14(9):1873-8.  26. Begon M, Scherrer SA, Coillard C, Rivard CH, Allard P. Three-dimensional vertebral wedging and pelvic asymmetries in the early stages of adolescent idiopathic scoliosis. Spine J. 2015;15(3):477-86.  27. Qiu XS, Wang ZW, Qiu Y, Wang WJ, Mao SH, Zhu ZZ et al. Preoperative pelvic axial rotation: a possible predictor for postoperative coronal decompensation in thoracolumbar/lumbar adolescent idiopathic scoliosis. Eur Spine J. 2013;22(6):1264-72.  28. Stępień A, Fabian K, Graff K, Podgurniak M, Wit A. An immediate effect of PNF specific mobilization on the angle of trunk rotation and the Trunk-Pelvis-Hip Angle range of motion in adolescent girls with double idiopathic scoliosis-a pilot study. Scoliosis Spinal Disord. 2017;12:29.  29. Dominiek Beckers MB. PNF in Practice: An Illustrated Guide. 5th ed. Berlin: Springer; 2021.  30. Hindle KB, Whitcomb TJ, Briggs WO, Hong J. Proprioceptive Neuromuscular Facilitation (PNF): Its Mechanisms and Effects on Range of Motion and Muscular Function. J Hum Kinet. 2012;31:105-13.  31. Sharman MJ, Cresswell AG, Riek S. Proprioceptive neuromuscular facilitation stretching : mechanisms and clinical implications. Sports Med. 2006;36(11):929-39. |
| --- |

**三、研究目标与研究内容**

| **1、研究目标**  **（列举出项目实施的目标）**  **1.1** 根据全脊柱正位X线上包括髋骨宽度的凹/凸比在内的骨盆及脊柱三维结构参数，评价“3+1”多维运动疗法在改善轻度青少年特发性脊柱侧凸曲线进展方面的有效性。  **1.2** 根据脊柱侧凸研究协会生活质量问卷SRS-22，评价“3+1”多维运动疗法在改善轻度青少年特发性脊柱侧凸患者生活质量方面的有效性。  **2、研究内容**  通过单中心、随机、对照研究，将轻度青少年特发性脊柱侧凸患者随机分配到试验组和对照组，试验组进行“3+1”多维运动疗法治疗，即Schroth治疗联合骨盆旋转矫正，对照组进行常规Schroth治疗，治疗时间24周。治疗结束后评估两组患者髋骨宽度的凹/凸比等骨盆及脊柱三维结构参数、SRS-22问卷等指标的差异，验证“3+1”多维运动疗法治疗在纠正躯干和骨盆三维结构畸形和提高生活质量方面的有效性，为青少年特发性脊柱侧凸的三维畸形矫正治疗提供新的临床证据。 |
| --- |

**四、研究方案**

| **1.研究方案**  **1.1 研究设计**  **1.1.1** 研究类型：单中心、随机、对照的临床应用研究。  **1.1.2** 研究假设：治疗24周后“3+1”多维运动治疗组，即骨盆旋转矫正治疗结合常规Schroth训练，比单独的Schroth治疗组在对改善轻度青少年特发性脊柱侧凸患者躯干及骨盆三维结构参数及生活质量方面更具优势。  **1.1.3** 随机和盲法：由一名统计学专家采用Microsoft Excel中的随机数函数程序进行区组随机数列生成，随机区组长度为4和6。按照随机数列，将患者以1:1的比例随机分配到实验组或对照组。随机分配序列被转移到一系列连续编号、密封、不透明的信封中，这些信封被存放在一个上锁的抽屉中，直到需要为止。当每个参与者正式进入试验时，分配信封在患者面前公开。  分组情况对收集和评估结果数据的医生和分析数据的统计学家均设盲。因物理治疗的特征无法对物理治疗师和患者设盲，然而，研究过程中要求治疗师和患者尽量不要透露他们的分组情况，以确保评估者的盲态。  **1.1.4** 治疗方案：  ①试验组：进行“3+1”多维运动治疗。治疗时间24周，前5周每周进行2次治疗，然后每两周进行1次治疗，共计20次治疗。  治疗内容：“3+1”多维运动治疗时间为90分钟，其中Schroth训练60分钟，包括矫正呼吸训练、腰部肌肉力量训练；骨盆旋转矫正治疗30分钟，包括骨盆周围肌肉对称性拉伸、骨盆轴向旋转矫正和核心稳定性训练。  Schroth治疗主要内容包括：矫正呼吸训练以及腰部肌肉力量训练两部分组成，训练方式为门诊一对一训练。（1）矫正呼吸训练：采取施罗斯“旋转对角呼吸”的方法。以腹式呼吸为基础，配合使弯弧凹侧充分打开的动作进行呼吸训练。吸气时通过治疗师手部辅助引导，引导患者有意识地吸气至弯弧的凹侧，吸气时间为4秒。呼气时发出“嘶”的声音，收紧腹肌，维持凹侧扩张状态，训练时间为20分钟。（2）腰部肌肉力量训练：在特定体位下，通过躯体动作激活、加强凹侧肌肉收缩，从而改善肌力，恢复脊柱两侧肌肉平衡，训练时间为40分钟。以左侧腰凸的肌肉圆柱训练为例，站立位时右侧下肢抬高30厘米，保持双下肢伸直，左手反手置于左髋部，右手搭肩，保持躯干左侧倾斜，结合呼吸激活右侧腰背部肌肉。吸气时，纵向延伸脊柱，将气体引导至凹侧，将凹侧扩张。呼气时胸部模块向左侧移动，同时保持右肩外旋状态 以改善剃刀背。右侧大腿向尾部伸展，降低右髋，同时将右足跟压向地板，激活右侧腰大肌，维持全身肌肉的等长收缩。配合矫正呼吸。10次/组，共3-5组。  骨盆旋转矫正治疗主要内容包括：（1）首先应用PNF拉伸技术纠正骨盆周围的短缩肌肉拉伸。在此之前通过Thomas试验等一系列物理测试评估连接骨盆和脊椎的肌肉张力，判断和识别骨盆周围缩短和张力增高的肌群，包括髋屈肌、髋伸展肌、髋内收肌、髋外展肌、侧旋肌，内侧旋转肌和腰方肌等。采用PNF的“保持-放松”技术拉伸缩短的肌群，治疗师辅助对抗目标肌肉进行等长收缩，收缩保持3-5秒，然后理疗师再拉伸10秒，以增加关节活动范围。整个过程重复3-4次。（2）其次，使用改良的PNF“保持-放松”技术对骨盆带进行10分钟的抗旋转治疗。根据患者的全脊柱X光片结果来确定骨盆带拉伸方向，骨盆旋转拉伸采用改良的双侧下肢屈曲-伸展模式。以骨盆顺时针旋转为例，双下肢屈髋屈膝贴近胸口，双膝向左倒向躯干左侧，同时由治疗师提供阻力，患者抵抗阻力进行向右向下伸展方向的等长收缩，收缩持续3-5秒后，治疗师继续将双膝向左侧拉伸10秒，以增加骨盆逆时针旋转的活动范围。整个过程重复3-4次，骨盆逆时针旋转的治疗原则相同。（3）最后，进行10分钟的核心稳定性训练，重点是增强脊柱周围肌肉的运动控制。  ②对照组：进行Schroth训练，治疗时间24周，前5周每周进行2次治疗，然后每两周进行1次治疗，共计20次治疗。每次治疗时间60分钟，治疗内容同试验组Schroth治疗部分。  **1.2 研究对象**  筛选于航天中心医院康复医学科就诊、诊断为青少年特发性脊柱侧弯的患者。根据2016年国际脊柱侧凸矫形与康复治疗协会（(Society on scoliosis Orthopaedic and Rehabilitation treatment，SOSORT）发布的关于青少年特发性脊柱侧凸矫形及康复治疗指南，医生评估患者脊柱侧凸情况并参考患者及其监护人意愿，将患者随机分配到试验组或对照组，所有参与研究的患儿及监护人均需签署知情同意书。  纳入标准：年龄10～18岁、主要弯弧Cobb角为10°～25°、Risser征0～5级、能够完成24周的治疗和评估。  排除标准：非特发性脊柱侧凸，即因神经肌肉系统疾病、先天性疾病及继发于其他疾病的脊柱侧凸；因下肢骨折、关节感染或关节炎、先天性疾病导致下肢不等长引起的继发性脊柱侧凸；既往脊柱手术史；曾经或正在佩戴脊柱矫形支具；认知功能障碍及系统性疾病等运动禁忌症。  **1.3 测试方法与指标**  **1.3.1 影像学测试指标：**站立位全脊柱正位X线测量参数。  a. 主弯Cobb角：找到向凹侧最倾斜的椎体以及向凸侧最倾斜的椎体，为上、下端椎，分别在上端椎上终板以及下端椎下终板做延长线并引垂线，两垂线夹角即为Cobb角。  b. 髋骨宽度的凹/凸比（ASIS-SI凹/凸比），即全脊柱正位X线中骶髂关节（SI）的下结节和髂前上棘（ASIS）垂直线间距的比值。  c. 冠状位骨盆倾斜（coronal pelvic tilt）；双侧髂嵴最高点水平切线与水平面的夹角。  d. 冠状位顶椎侧移（apical vertebral translation，AVT）：最大弯弧顶椎中心与骶骨中垂线之间的线性距离，以毫米为单位。  e. 顶椎椎体旋转（apical vertebral rotation，AVR）：通过Nash-Moe 5级分级法评估顶端椎骨旋转程度，其中0级表示顶端椎骨没有旋转，IV级表示严重旋转。  由一名固定医师通过Image-Pro Plus 6.0软件进行测量，共进行三次测量，计算三次测量的平均值用于分析。  **1.3.2 体表测试指标**  躯干旋转角度（axial trunk rotation angle，ATR）：受试对象尽可能的暴露上半身，做Adams前屈试验，手部自然下垂，由固定医师使用脊柱侧凸测量仪Scoliometer于患者正后方测量躯干倾斜角度，康复师双手持Scoliomter尺，将尺子0°中心置于脊柱棘突位置，视线与尺子水平，沿棘突从上之下缓慢移动，待稳定后读取度数。度数越高代表躯干旋转越严重。由一名固定医师进行体表测量，共进测量三次，采用三次测量的平均值用于分析。  **1.3.3 日常生活质量评价指标**  脊柱侧凸研究协会生活质量问卷（Scoliosis Research Society-22，SRS-22）：评估脊柱侧凸患者的功能活动、疼痛、自我形象、心理状况及对治疗的满意程度。问卷包括22个项目，各个项目均是1～5的5点评估，5分代表极好，1分代表极差。问卷涉及以下5个因素：功能活动 (项目5，9，12，15，18)，疼痛 (项目1，2，8，11，17)，**自我形象** (项目4，6，10，14，19)，心理健康 (项目3，7，13，16，20)，和对治疗的满意程度 (项目21，22)。总分110分，分值越高代表生活质量越好。  **1.4 评价指标和随访计划**  （1）主要评价指标：髋骨宽度的凹/凸比（ASIS-SI凹/凸比）  （2）次要评价指标：  ① 主要弯弧Cobb角度；  ② 轴向躯干旋转角度（ATR）；  ③ 椎体旋转（apical vertebral rotation，AVR）；  ④ 脊柱侧凸研究协会生活质量问卷—自我形象评分；  ⑤ 冠状骨盆倾斜（coronal pelvic tilt）；  ⑥ 冠状位顶椎侧移（apical vertebral translation，AVT）。  各指标由不参与患者筛选和治疗的固定医师进行评估。  （3）随访计划：随访由固定的康复治疗师进行主动电话随访并预约门诊复查。治疗结束后进行轴向躯干旋转角度（ATR）、健康相关生活质量问卷(SRS-22)得分等临床评估；复查站立位全脊柱正位X线。在随访过程中，如果发现脊柱外形的明显恶化需要立即进行站立位全脊柱正位X线检查。  **不良事件处理**：在治疗过程中，如果发现脊柱外形的明显变化或有持续疼痛（疼痛超过1周不缓解），需要立即停止治疗，该病例将被撤出研究并从主要结局分析中排除。  **1.5 样本量的确定依据**  本研究为随机平行对照设计，试验组和对照组按照1:1比例随机入组，主要结局指标为髋骨宽度的凹/凸比。样本量计算基于2021年6月至2021年11月期间接受同一治疗方案的12名轻度AIS患者的髋骨宽度的凹/凸比：基线凹凸比平均值±标准差（SD）为（93±3.2）%；24周时，实验组的平均凹凸比增加了3%，对照组没有变化；设*α*为0.05（双侧），*β*为0.2，以检测组间差异；预期失访率为10%。基于以上假设，预估总样本量为42例，其中试验组21例，对照组21例。  **1.6 统计分析方法**  统计分析根据意向性分析（intention to treat，ITT）原则，分析包括所有随机化并在入组后接受至少一次治疗的受试者，遵循末次观察值结转法（last observation carried forward，LOCF）处理缺失数据。  数据采用SPSS 22.0统计软件分析处理。人口学指标、基线数据采用Shapiro-Wilk检验确认变量的分布情况。如符合正态分布，计量资料以均数±标准差（‾x±s）进行描述以比较组间的均衡性，采用单因素协方差分析比较组间差异；计数资料以频数/百分比进行描述，采用卡方检验χ^2^进行差异性检验；非正态分布的计量资料以中位数（上下四分位数）表示，组间比较采用Wilcoxon秩和检验。主要评价指标采用协方差分析（analysis of covariance，ANCOVA）进行评估，以基线值为协变量，次要评价指标采用ANCOVA和卡方检验进行分析，采用Pearson相关和点二列相关评估主要结果和其他次要结果测量之间的相关性。统计分析结果采用‾x（95%置信区间，95% CI），所有统计分析基于双侧假设检验，以*α*=0.05为检验水准。  **1.7 数据采集和管理**  ①数据采集：采取病例报告表（CRF）的记录方式，由临床研究者及时、准确地将CRF中的数据录入数据库中。严格遵循研究计划方案完成数据采集，保证数据收集准确、及时、完整。  ②数据管理：由监察员对病历录入内容与原始资料进行核查，监查员根据所录入的所有数据提出疑问，录入人员或研究者对问题做解答，并修改错误数据，所有修改记录均保存在CRF中。  ③所有数据审核无误后，监察人员对数据进行锁定、保存。  **2.技术路线**  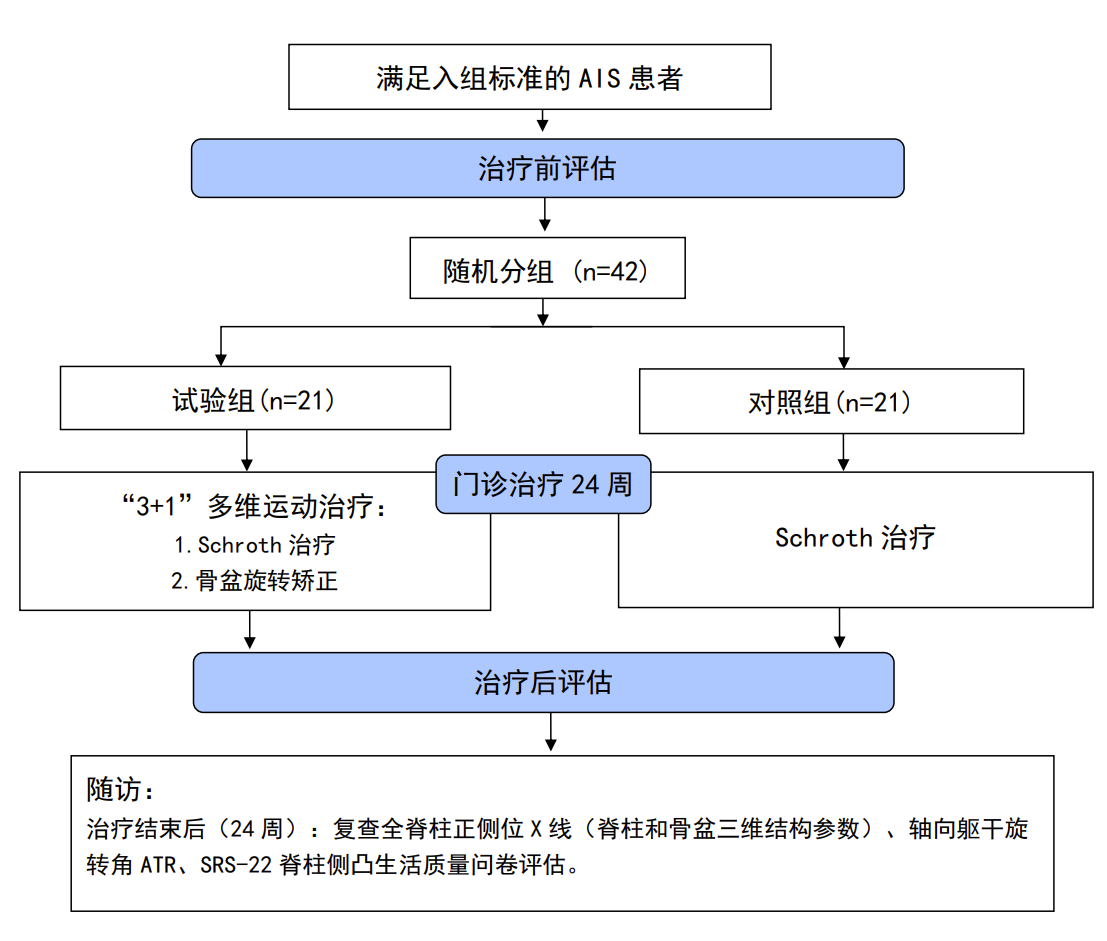  图2. 研究技术路线图  **3.临床研究过程中质量控制措施**  ⑴遵循研究计划方案：临床试验开始前，项目负责人对研究者进行试验方案的培训，有序规范地开展试验，严格遵循研究计划方案完成治疗。  ⑵知情同意书的签署：保障患者权益，保证知情同意书及时签署。  ⑶试验方案的依从性：定期随访；健康宣教，使家长或监护人充分发挥监督作用；保持治疗师与患者的及时沟通等形式提高患者依从性。  ⑷所有数据的记录与报告的质量控制：对结局指标测量流程进行培训，并针对各个指标由专门医生进行测量；设立监察人员，保证研究方案的所有内容都得到严格遵守，并对原始资料进行监查以确保与CRF上的内容一致。  ⑸不良事件/反应：确认所有不良事件记录在案，严重不良事件使病人得到及时的处理，并在规定时间内做出报告并记录在案。  ⑹资料的归档保存：病例报告资料及时归档，并由专人专柜保管。 |
| --- |
