# Supplementary material for: Pelvic rotation correction combined with Schroth exercises for pelvic and spinal deformities in mild adolescent idiopathic scoliosis: A randomized controlled trial
Source: PLoS One. 2024 Jul 30;19(7):e0307955. doi: 10.1371/journal.pone.0307955 (PMC11288462; doi:10.1371/journal.pone.0307955)
Supplement: S4 File — (DOCX) [file pone.0307955.s005.docx]

| **Project Number** |
| --- |
| HP2022-50-507005 |

**Hygiene and Health Development Scientific Research Fostering Plan of Haidian District Beijing**

**Program mission statement**

Project name: The Impact of "3+1" Multidimensional Therapeutic Exercises on the Progression of Adolescent Idiopathic Scoliosis

Type of specialty: Chinese medicine ( ) Western medicine ( √ ) Management ( )

Conversion projects: Yes ( ) No (√)

Application unit: Aerospace Center Hospital

Applicant: Yafei Zhang

Contact: Yafei Zhang

Tel: 13436639195

Starting date: January 2022 to December 2023

**I. Basic project information**

| **Project Information** | Project name | The impact of "3+1" multidimensional therapeutic exercises on the progression of adolescent idiopathic scoliosis |
| --- | --- | --- |
|  | Keywords (≤5) | Pelvis; Rotation; Scoliosis; Adolescent; Physical Therapy Modalities |
|  | Project Type | □ Basic Research 🗹 Clinical Research |
| **Project Summary (400 word limit):** | | |
| Adolescent idiopathic scoliosis is the most common three-dimensional structural deformity of the spine in adolescents, and while scoliosis-specific physiotherapy such as Schroth exercise in controlling scoliosis progression has been confirmed, there is insufficient evidence to support its effectiveness for axial trunk deformity. Some studies have suggested that axial rotation of the pelvis may be involved in the progression of idiopathic scoliosis, and correction of pelvic rotation may have a therapeutic effect on the improvement of three-dimensional deformities of the trunk. However, there is a lack of high-quality clinical studies on the treatment of three-dimensional scoliosis deformities by correction of pelvic axial rotation. Therefore, we plan to conduct a single-center, randomized, controlled trial to evaluate the efficacy of "3+1" multidimensional exercise therapy (including Schroth therapy and pelvic asymmetry correction therapy) in adolescents with mild idiopathic scoliosis. Patients will be randomized to receive "3+1" multidimensional exercise therapy or Schroth therapy alone for 24 weeks, and three-dimensional structural parameters of the pelvis and spine, such as pelvic rotation parameters, Cobb angle, trunk rotation angle, and self-image perception will be compared between the two groups at the end of treatments. This study aims to verify the effectiveness of "3+1" multidimensional exercise therapy in improving the three-dimensional deformities of the trunk and acceptance of self-image in adolescents with mild idiopathic scoliosis. The study will provide clinical evidence to support the treatment strategy for idiopathic scoliosis in adolescents. | | |

**II.** **Rationale for topic selection**

| **1. Research Objectives and Significance**  Adolescent idiopathic scoliosis (AIS) is a three-dimensional structural deformity affecting the spine and trunk that typically arises in healthy children or adolescents during growth and development stages. It usually manifests as scoliosis with axial rotation of the spine and altered sagittal curves[1, 2] The International Scoliosis Research Society (SRS) recognizes a Cobb's angle of ≥10°, measured on standing posterior-anterior full-spine radiographs, as a diagnostic criterion for scoliosis[3]. The prevalence of scoliosis in the world is approximately 2% in Asia and some parts of China [4] Adolescent idiopathic scoliosis (AIS) usually develops before skeletal maturity, particularly during pre-puberty. Girls between 10-14 years and boys between 12-16 years of age have a high risk of onset. Additionally, children and adolescents with immature skeletons during their peak growth spurts are at a very high risk of progression [5-7]. Spinal deformities progress rapidly and affect physical appearance during growth. They can also have negative effects on adolescent mental health and quality of life. Large-angle scoliosis can result in pulmonary complications and pain, which increases the risk of health disorders and surgery in adulthood [8-10].  The etiology and pathogenesis of AIS are unclear. It has been suggested that the pelvis may be an important factor in the development and progression of scoliosis [11-14]. Jeff L. Gum [15] suggested that axial pelvic rotation may be associated with thoracic scoliosis, and in cases of AIS with compensatory thoracolumbar or lumbar curves, axial pelvic rotation appears to align with the primary thoracic curve to maintain overall pelvic balance. Xu-Sheng Qiu et al. found that patients with AIS have both spinal and axial pelvic rotation, resulting in an uneven concave/convex hip width ratio [16].  The pelvis serves as the origin and insertion point for all abdominal and iliopsoas muscles; therefore, during rapid pubertal development, any imbalance in abdominal muscle structure and strength will affect pelvic balance [17]. Research has indicated that relative thickness of abdominal muscles is more asymmetrical in individuals with AIS than in healthy adolescents [17-19]. This asymmetry may be linked to pelvic rotation, which could, in turn, add to the longitudinal axis rotation of scoliosis. Therefore, possible interventions in pelvic rotation may impact the structure of peripheral attachment muscle groups, including the abdominal muscles. Conversely, pelvic asymmetry is an important factor in the development of scoliosis; pelvic symmetry is crucial both in its role of connecting the spine to the lower extremities and in ensuring symmetry of bony structures in scoliosis and the ability to balance the lower extremities [20].  **2. Analysis of the Current Research Status and Existing Issues at Home and Abroad**  The main goal of conservative treatment of AIS is to halt and slow the progression of the Cobb angle, in addition to correcting the three-dimensional trunk deformity associated with scoliosis. The Cobb angle is considered the gold standard for assessing scoliosis deformity and is neither a unique nor the only decisive parameter in patients with mild AIS. In recent years, the efficacy of scoliosis-specific exercise therapy in patients with mild to moderate AIS, as represented by Schroth (Germany), has been supported by numerous high-level clinical evidence, and the coronal plane deformity in patients with mild to moderate scoliosis has been well controlled by scoliosis-specific physiotherapy [21-23], but there is insufficient evidence to support the effect on the axial deformity of the trunk.  Axial pelvic rotation may be associated with spinal deformities such as vertebral rotation of the thoracic or thoracolumbar spine [15, 24, 25]. Begon et al. suggested that pelvic asymmetry is associated with the progression of scoliosis [26]. Qiu et al. also suggested that AIS patients with preoperative axial pelvic rotation are at greater risk of postoperative coronal imbalance [27]. In addition, more studies are now focusing on the treatment of three-dimensional trunk deformity, and some studies have shown that correction of pelvic rotation has a therapeutic effect on improving three-dimensional structural trunk deformity.  In a randomized controlled trial, Abdel-azie et al. demonstrated that a 10-week combination of hippotherapy and Schroth exercise improved postural asymmetries such as scoliosis angle, axial pelvic rotation, and axial vertebral rotation in patients with AIS[20]. The authors suggested that the underlying mechanism for the improvement of three-dimensional spinal deformity with horse riding may be that the horse generates three-dimensional motion similar to that of the human pelvis during walking, and the horse's motion generates rotational motion along the longitudinal axis in the pelvis of the scoliosis patient riding on it, which in turn generates upward spinal curvature and rotation. However, hippotherapy is a relatively expensive method and may not be suitable for most Chinese adolescents.  Proprioceptive neuromuscular facilitation (PNF) stretching techniques are commonly used in patients with movement problems, including axial asymmetry of the pelvis in AIS, to improve rotational range of motion caused by imbalanced muscles connecting the pelvis to the spine [28-31]. An interventional study conducted by Stypien et al. demonstrated a short-term improvement in trunk rotation angles in girls with adolescent idiopathic scoliosis, suggesting that PNF stretching may be an effective method for correcting trunk and pelvic asymmetry in the transverse plane of AIS [28], but the lack of imaging evidence in this study and the absence of a control group limit the extrapolation of the findings to clinical applications.  **3. Application direction or application perspective**  Previously, our research team conducted a pilot study of the "3+1" multidimensional exercise treatment for adolescent idiopathic scoliosis at our research center, i.e., the incorporation of pelvic rotation correction into Schroth scoliosis treatment, which combines the correction of three-dimensional structural deformities of the pelvis and trunk in patients with AIS (Figure 1). In this pilot study, we treated 12 patients with adolescent idiopathic scoliosis with a mean age of 13.6 years and a Cobb angle of 10° to 25° for 24 weeks. The evaluation at the end of the treatment showed that patients with AIS who received Schroth treatment combined with pelvic rotation correction had more significant improvement in pelvic axial symmetry, trunk rotation angle index, vertebral axial rotation, and Cobb angle, which provided strong results to support our research work. Therefore, this study aims to use a prospective randomized controlled trial design to investigate the effectiveness of "3+1" multidimensional exercise therapy for the treatment of mild adolescent idiopathic scoliosis involving three-dimensional structural deformities of the trunk, and to provide clinical evidence for the therapeutic strategy of adolescent idiopathic scoliosis.  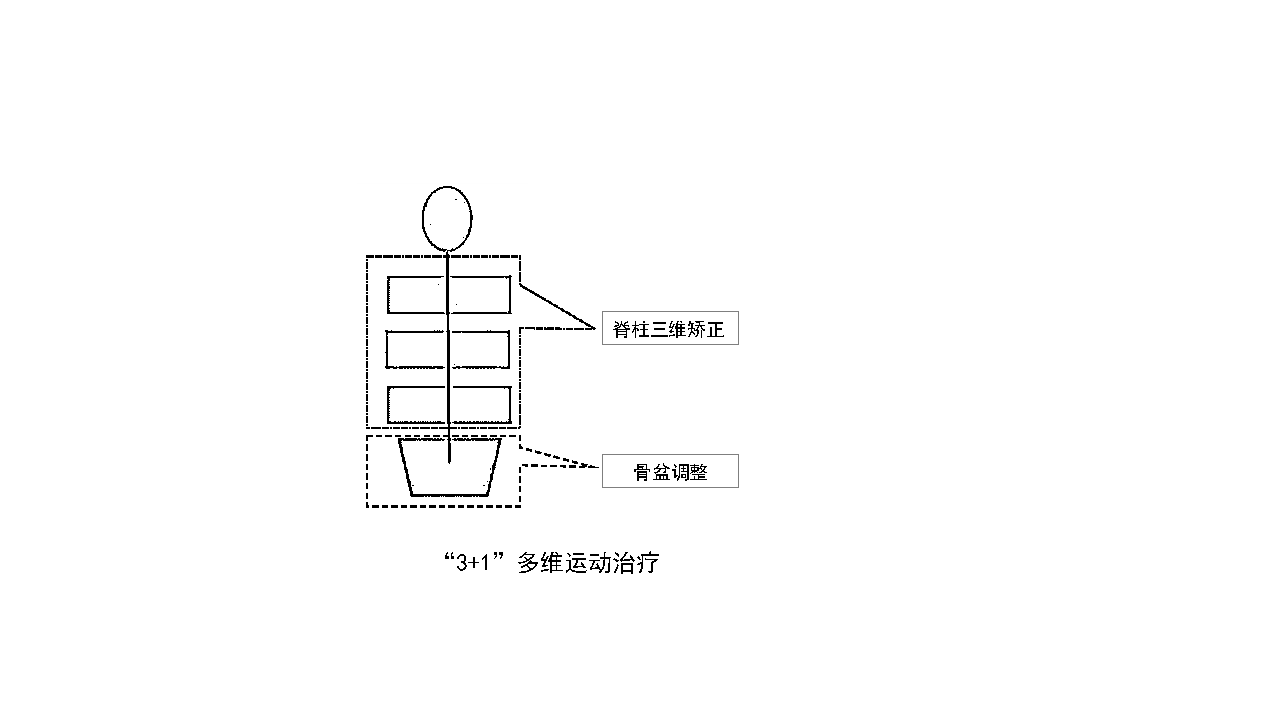  Figure 1. Schematic diagram of the "3+1" multidimensional movement therapy  Note: "3"-spinal 3D correction (Schroth treatment); "1"-pelvic asymmetry correction  **Reference:**  **(Reference format: paper: author, title, journal title, year, volume (issue), starting and ending page numbers; Monographs: author, title, publisher, year.)**  1. Negrini S, Donzelli S, Aulisa AG, Czaprowski D, Schreiber S, de Mauroy JC et al. 2016 SOSORT guidelines: orthopaedic and rehabilitation treatment of idiopathic scoliosis during growth. Scoliosis Spinal Disord. 2018;13:3.  2. Hresko MT. Clinical practice. Idiopathic scoliosis in adolescents. N Engl J Med. 2013;368(9):834-41.  3. Weinstein SL, Dolan LA, Cheng JC, Danielsson A, Morcuende JA. Adolescent idiopathic scoliosis. Lancet. 2008;371(9623):1527-37.  4. Zhang H, Guo C, Tang M, Liu S, Li J, Guo Q et al. Prevalence of scoliosis among primary and middle school students in Mainland China: a systematic review and meta-analysis. Spine (Phila Pa 1976). 2015;40(1):41-9.  5. Loncar-Dusek M, Pecina M, Prebeg Z. A longitudinal study of growth velocity and development of secondary gender characteristics versus onset of idiopathic scoliosis. Clin Orthop Relat Res. 1991(270):278-82.  6. Busscher I, Kingma I, de Bruin R, Wapstra FH, Verkerke GJ, Veldhuizen AG. Predicting the peak growth velocity in the individual child: validation of a new growth model. Eur Spine J. 2012;21(1):71-6.  7. Lonstein JE, Carlson JM. The prediction of curve progression in untreated idiopathic scoliosis during growth. J Bone Joint Surg Am. 1984;66(7):1061-71.  8. Di Felice F, Zaina F, Donzelli S, Negrini S. The Natural History of Idiopathic Scoliosis During Growth: A Meta-Analysis. Am J Phys Med Rehabil. 2018;97(5):346-56.  9. Koumbourlis AC. Scoliosis and the respiratory system. Paediatr Respir Rev. 2006;7(2):152-60.  10. Tones M, Moss N, Polly DW, Jr. A review of quality of life and psychosocial issues in scoliosis. Spine (Phila Pa 1976). 2006;31(26):3027-38.  11. Karski T. Etiology of the so-called "idiopathic scoliosis". Biomechanical explanation of spine deformity. Two groups of development of scoliosis. New rehabilitation treatment; possibility of prophylactics. Stud Health Technol Inform. 2002;91:37-46.  12. Fiala L. [Etiology of the so-called idiopathic scoliosis]. Bratisl Lek Listy. 1954;34(12):1452-6.  13. Saji MJ, Upadhyay SS, Leong JC. Increased femoral neck-shaft angles in adolescent idiopathic scoliosis. Spine (Phila Pa 1976). 1995;20(3):303-11.  14. Burwell RG, Cole AA, Cook TA, Grivas TB, Kiel AW, Moulton A et al. Pathogenesis of idiopathic scoliosis. The Nottingham concept. Acta Orthop Belg. 1992;58 Suppl 1:33-58.  15. Gum JL, Asher MA, Burton DC, Lai SM, Lambart LM. Transverse plane pelvic rotation in adolescent idiopathic scoliosis: primary or compensatory? Eur Spine J. 2007;16(10):1579-86.  16. Qiu XS, Zhang JJ, Yang SW, Lv F, Wang ZW, Chiew J et al. Anatomical study of the pelvis in patients with adolescent idiopathic scoliosis. J Anat. 2012;220(2):173-8.  17. Doran M, Öneş K, Terzibaşioğlu AM, Çinar Ç, Ata İ. Ultrasonographic evaluation of abdominal muscle thickness symmetry in adolescent idiopathic scoliosis: a case-controlled study. Eur J Phys Rehabil Med. 2021;57(6):968-76.  18. Linek P, Saulicz E, Kuszewski M, Wolny T. Ultrasound Assessment of the Abdominal Muscles at Rest and During the ASLR Test Among Adolescents With Scoliosis. Clin Spine Surg. 2017;30(4):181-6.  19. Ma CZ, Ren LJ, Cheng CL, Zheng YP. Mapping of Back Muscle Stiffness along Spine during Standing and Lying in Young Adults: A Pilot Study on Spinal Stiffness Quantification with Ultrasound Imaging. Sensors (Basel). 2020;20(24).  20. Abdel-Aziem AA, Abdelraouf OR, Ghally SA, Dahlawi HA, Radwan RE. A 10-Week Program of Combined Hippotherapy and Scroth's Exercises Improves Balance and Postural Asymmetries in Adolescence Idiopathic Scoliosis: A Randomized Controlled Study. Children (Basel). 2021;9(1).  21. Kuru T, Yeldan İ, Dereli EE, Özdinçler AR, Dikici F, Çolak İ. The efficacy of three-dimensional Schroth exercises in adolescent idiopathic scoliosis: a randomised controlled clinical trial. Clin Rehabil. 2016;30(2):181-90.  22. Liu D, Yang Y, Yu X, Yang J, Xuan X, Yang J et al. Effects of Specific Exercise Therapy on Adolescent Patients With Idiopathic Scoliosis: A Prospective Controlled Cohort Study. Spine (Phila Pa 1976). 2020;45(15):1039-46.  23. Negrini S, Donzelli S, Negrini A, Parzini S, Romano M, Zaina F. Specific exercises reduce the need for bracing in adolescents with idiopathic scoliosis: A practical clinical trial. Ann Phys Rehabil Med. 2019;62(2):69-76.  24. Zhao Y, Qi L, Yang J, Zhu X, Yang C, Li M. Factors affecting pelvic rotation in idiopathic scoliosis: Analysis of 85 cases in a single center. Medicine (Baltimore). 2016;95(46):e5458.  25. Wang ZW, Wang WJ, Sun MH, Liu Z, Zhu ZZ, Zhu F et al. Characteristics of the pelvic axial rotation in adolescent idiopathic scoliosis: a comparison between major thoracic curve and major thoracolumbar/lumbar curve. Spine J. 2014;14(9):1873-8.  26. Begon M, Scherrer SA, Coillard C, Rivard CH, Allard P. Three-dimensional vertebral wedging and pelvic asymmetries in the early stages of adolescent idiopathic scoliosis. Spine J. 2015;15(3):477-86.  27. Qiu XS, Wang ZW, Qiu Y, Wang WJ, Mao SH, Zhu ZZ et al. Preoperative pelvic axial rotation: a possible predictor for postoperative coronal decompensation in thoracolumbar/lumbar adolescent idiopathic scoliosis. Eur Spine J. 2013;22(6):1264-72.  28. Stępień A, Fabian K, Graff K, Podgurniak M, Wit A. An immediate effect of PNF specific mobilization on the angle of trunk rotation and the Trunk-Pelvis-Hip Angle range of motion in adolescent girls with double idiopathic scoliosis-a pilot study. Scoliosis Spinal Disord. 2017;12:29.  29. Dominiek Beckers MB. PNF in Practice: An Illustrated Guide. 5th ed. Berlin: Springer; 2021.  30. Hindle KB, Whitcomb TJ, Briggs WO, Hong J. Proprioceptive Neuromuscular Facilitation (PNF): Its Mechanisms and Effects on Range of Motion and Muscular Function. J Hum Kinet. 2012;31:105-13.  31. Sharman MJ, Cresswell AG, Riek S. Proprioceptive neuromuscular facilitation stretching : mechanisms and clinical implications. Sports Med. 2006;36(11):929-39. |
| --- |

**III. Research Objectives and Scope**

| **1. Research objectives**  **(List the objectives of project implementation)**  **1.1** To evaluate the efficacy of "3+1" multidimensional exercise therapy in improving the progression of mild adolescent idiopathic scoliosis curves based on three-dimensional structural parameters of the pelvis and spine, including the concavity/convexity ratio of the hipbone width, on standing full-spine posteroanterior radiographs.  **1.2** To evaluate the effectiveness of "3+1" multidimensional exercise therapy in improving quality of life in patients with mild adolescent idiopathic scoliosis as assessed by the Scoliosis Research Society Quality of Life Questionnaire SRS-22.  **2. Research scope**  In a single-center, randomized, controlled trial, patients with mild adolescent idiopathic scoliosis will be randomized to an experimental group and a control group. The experimental group will be treated with "3+1" multidimensional kinesiology, i.e. Schroth therapy combined with pelvic rotation correction, and the control group will be treated with conventional Schroth therapy for 24 weeks. At the end of the treatment, the differences in the three-dimensional structural parameters of the pelvis and spine, such as the concave/convex ratio of the width of the hip bone, and the SRS-22 questionnaire were evaluated to verify the effectiveness of the "3+1" multidimensional therapy in correcting the three-dimensional structural deformities of the trunk and pelvis and improving the quality of life, and to provide a new method for correcting the three-dimensional deformities of idiopathic scoliosis in adolescents. This study will provide new clinical evidence for the physiotherapy of mild adolescent idiopathic scoliosis. |
| --- |

**IV. Research program**

| **1. Research program**  **1.1 Study design**  **1.1.1** Type of study: single-center, randomized, controlled clinical application study.  **1.1.2** Hypothesis: After 24 weeks of treatment, the "3+1" multidimensional exercise therapy group, i.e., pelvic rotation therapy combined with conventional Schroth training, will be more beneficial than the Schroth therapy group alone in improving three-dimensional structural parameters of the trunk and pelvis and quality of life in patients with mild adolescent idiopathic scoliosis.  **1.1.3** Randomization and Blinding: A statistician uses the Random Number Function program in Microsoft Excel to generate random number series for the zones, which are 4 and 6 in length. Patients are randomly assigned to either the experimental or control group in a 1:1 ratio according to the random number series. The random assignment sequence is transferred to a series of consecutively numbered, sealed, opaque envelopes that are stored in a locked drawer until needed. The allocation envelopes are disclosed in front of the patient when each participant is formally enrolled in the study.  The allocation is blinded to both the physicians who collect and assess the outcome data and the statisticians who analyze the data. It is not possible to blind physiotherapists and patients due to the nature of physiotherapy, but therapists and patients are asked to try not to disclose their subgroup allocation during the study to ensure blinding of the assessors.  **1.1.4** Treatment programs:  ① Experimental group: "3+1" multidimensional exercise treatment. Treatment period is 24 weeks, with two sessions per week for the first 5 weeks and then one session every 2 weeks for a total of 20 sessions.  Treatment Content: "3+1" multidimensional exercise treatment time is 90 minutes, comprising Schroth training for 60 minutes, which includes corrective breathing training, lumbar muscle strength training; pelvic rotation correction treatment for 30 minutes, which includes symmetrical stretching of the peripheral pelvic muscles, correction of axial rotation of the pelvis, and core stability training.  Schroth treatment mainly consists of two parts: corrective breathing training and lumbar muscle strength training, the training mode is outpatient one-on-one training. (1) Corrective breathing training: The Schroth method of "rotational diagonal breathing" is adopted. It is based on abdominal breathing, with the effect that the concave side of the curved arc is fully opened for breathing training. Inhalation is guided by the therapist's hand, guiding the patient to consciously inhale into the concave side of the curved arc, and the inhalation time is 4 seconds. When exhaling, a "hissing" sound is made to tighten the abdominal muscles and maintain the expansion of the concave side, and the training time is 20 minutes. (2) Lumbar muscle strength training: In a certain position, activate and strengthen the muscle contraction on the concave side by body movement, so as to improve the muscle strength and restore the balance of the muscles on both sides of the spine; the training time is 40 minutes. Taking the example of the muscle cylinder training of the left lumbar convex side, in the standing position, raise the right lower limb by 30 centimeters, keep both lower limbs straight, place the left-hand backhand on the left hip, put the right hand on the shoulder, keep the torso tilted to the left side, and activate the right lumbar and back muscles in combination with breathing. On inhalation, lengthen the spine to direct the gas to the concave side and expand the concave side. On exhalation, move the thoracic module to the left while keeping the right shoulder externally rotated to improve the razorback. Extend the right thigh caudally and lower the right hip while pressing the right heel toward the floor to activate the right psoas major and maintain isometric contraction throughout the body. Work with corrective breathing. 10 repetitions/set for 3-5 sets.  The main components of pelvic asymmetry correction therapy include (1) first applying the PNF stretching technique to correct the shortened muscle tone around the pelvis. This is preceded by a series of physical tests, such as the Thomas test, to assess the tone of the muscles connecting the pelvis to the spine, and to determine and identify the shortened and hypertonic muscles around the pelvis, including the hip flexors, hip extensors, hip adductors, hip abductors, lateral rotators, medial rotators, and lumbar muscles, etc. The PNF "hold-relax" technique is used to stretch the shortened muscles around the pelvis. Shortened muscles are stretched using the PNF "hold-relax" technique, where the therapist assists with isometric contractions against the target muscle, holding the contraction for 3-5 seconds, and then the therapist stretches the muscle for another 10 seconds to increase the range of motion of the joint. This process is repeated 3-4 times. (2) Next, the pelvic girdle is treated with 10 minutes of anti-rotation using a modified PNF "hold-relax" technique. The direction of pelvic girdle stretching was determined based on the patient's full spine radiographs, and pelvic rotation stretching was performed using a modified bilateral lower extremity flexion-extension pattern. Using clockwise pelvic rotation as an example, both lower extremities were flexed at the hips and knees close to the chest, and both knees were flexed to the left toward the left side of the torso while resistance was provided by the therapist, and the patient resisted the resistance by performing isometric contractions in the direction of rightward downward stretching and the contractions lasted for 3-5 seconds, after which the therapist continued to stretch the knees to the left for 10 seconds to increase the range of motion for the counterclockwise rotation of the pelvis. The entire process was repeated 3-4 times using the same principles of treatment for counterclockwise pelvic rotation. (3) Finally, a 10-minute core stability exercise is performed, focusing on increasing motor control of the muscles around the spine.  ② Control Group: Schroth training was performed for 24 weeks, with 2 sessions per week for the first 5 weeks and then 1 session every 2 weeks for a total of 20 sessions. Each treatment lasted 60 minutes, and the treatment content was the same as the Schroth treatment part of the experimental group.  **1.2 Study population**  Patients attending the Department of Rehabilitation Medicine at the Aerospace Center Hospital and diagnosed with adolescent idiopathic scoliosis are evaluated. According to the guidelines on orthopaedic and rehabilitative treatment of adolescent idiopathic scoliosis issued by the International Society on Scoliosis Orthopaedic and Rehabilitation Treatment (SOSORT) in 2016, One rehabilitation physician assesses the patients' scoliosis and refers to the wishes of the patients and their guardians, and randomly assigns the patients to either the experimental group or the control group, and all children and their guardians who participate in the study are required to sign an informed consent form.  Inclusion criteria: age 10 to 18 years, Cobb angle of 10° to 25° in the major curve, Risser's sign grade 0 to 5, and ability to complete 24 weeks of treatment and evaluation.  Exclusion criteria: non-idiopathic scoliosis, i.e., scoliosis due to neuromuscular disorders, congenital disorders, and secondary to other disorders; secondary scoliosis due to lower extremity fractures, joint infections, or arthritis; and lower extremity inequality due to congenital disorders; history of previous spinal surgery; previous or current orthopedic spinal braces; and contraindications to exercise such as cognitive dysfunction and systemic diseases.  **1.3 Test methods and indicators**  **1.3.1 Radiographic Index:** Standing full-spine posteroanterior radiographic measurement parameters.  a. Cobb angle of the primary curve: Find the most inclined vertebrae to the concave side and the most inclined vertebrae to the convex side for the upper and lower end vertebrae, respectively, and make extension lines and draw vertical lines in the upper end plate of the upper end vertebrae and the lower end plate of the lower end vertebrae, and the angle of the two vertical lines will be the Cobb angle.  b. The concavity/convexity ratio of the width of the hip bone (ASIS-SI concavity/convexity ratio), i.e., the ratio of the distance between the vertical lines of the inferior tubercle of the sacroiliac joint (SI) and the anterior superior iliac spine (ASIS) in the full spine radiograph.  c. Coronal Pelvic Tilt (CPT); the angle between the horizontal tangent line at the highest point of the iliac crests bilaterally and the horizontal plane.  d. Coronal apical vertebral translation (AVT); the linear distance in millimeters between the center of the vertebral apex of the maximum arc of curvature and the mid-sacral plumb line.  e. Apical Vertebral Rotation (AVR): The degree of apical vertebral rotation was assessed using the Nash-Moe 5-grade scale, with grade 0 indicating no rotation of the apical vertebrae and grade IV indicating severe rotation.  Measurements are performed by a senior physician using Image-Pro Plus 6.0 software, three measurements are taken, and the average of the three measurements is calculated for analysis.  **1.3.2 Anthropometric indices**  Angle of Trunk Rotation (ATR): With the patient's upper body exposed as much as possible, the Adams forward bending test is performed with the hands naturally hanging, the physician uses the scoliometer to measure the angle of trunk inclination in the back of the patient, and the physician holds the scoliometer ruler with both hands and places the ruler at the 0° center of the spine at the spinous process of the spine. Holding the Scoliometer ruler in both hands, the physician places the 0° center of the ruler on the spinous process of the spine, with the line of sight at the level of the ruler, and slowly moves the ruler along the spinous process from top to bottom, and then reads the number of degrees after it has stabilized. The higher the degree, the greater the rotation of the trunk. Measurements are performed by a physician. A total of three measurements are taken, and the average of the three measurements is calculated for analysis.  **1.3.3 Assessment of the quality of daily life**  Scoliosis Research Society Quality of Life Questionnaire (Scoliosis Research Society-22, SRS-22): It is used to assess functional activity, pain, self-image, psychological status, and satisfaction with treatment in patients with scoliosis. The questionnaire consists of 22 items, and each item is scored on a 5-point scale from 1 to 5, with a score of 5 representing excellent and 1 representing very poor. The questionnaire addresses the following 5 factors: functional activity (items 5, 9, 12, 15, 18), pain (items 1, 2, 8, 11, 17), self-image (items 4, 6, 10, 14, 19), psychological well-being (items 3, 7, 13, 16, 20), and satisfaction with treatment (items 21, 22). The total score is 110, with higher scores representing better quality of life.  **1.4** **Evaluation indicators and plan for follow-up**  (1) Primary Outcome Measure: concavity/convexity ratio of the width of the hip bone (ASIS-SI concavity/convexity ratio)  (2) Secondary Outcome Measure:  ① Cobb angle of the primary curve;  ② Axial Trunk Rotation (ATR);  ③ Apical vertebral rotation (AVR);  ④ Scoliosis Research Society-22 (SRS-22)—Self-Image Score;  ⑤ Coronal pelvic tilt (CPT);  ⑥ Apical vertebral translation (AVT).  Indicators are assessed by physicians who are not involved in the screening and treatment of patients.  (3) Follow-up plan: The follow-up is conducted by a rehabilitation therapist, who makes active telephone calls and schedules outpatient evaluations. At the end of treatment, clinical assessments such as axial trunk rotation (ATR) and scores on the Health-Related Quality of Life Questionnaire (SRS-22) will be performed; and full spine X-rays will be reviewed. During the follow-up, if a significant deterioration in back shape is noted, the X-rays should be taken immediately.  TREATMENT OF ADVERSE EVENTS: If any significant change in the shape of the spine or persistent pain (pain unrelieved for more than 1 week) requiring immediate discontinuation of treatment is noted during the course of treatment, the case is withdrawn from the study and excluded from the analysis of the primary outcome.  **1.5 Basis for determining sample size**  The study is a randomized, parallel controlled design with 1:1 randomization of the treatment and control groups and the primary outcome measure is the concave/convex ratio of hip bone width. Sample size calculations are based on the concave/convex ratio of hip width in 12 patients with mild AIS who received the same treatment regimen between June 2021 and November 2021. The baseline concave/convex ratio mean ±standard deviation (SD) is (93± 3.2) %; the mean concave/convex ratio at 24 weeks increased by 3% in the experimental group and remain unchanged in the control group; α is set at 0.05 (bilateral) and β at 0.2 to detect between-group differences; and the expected loss to follow-up rate is 10%. Based on these assumptions, the predicted total sample size is 42 cases, with 21 cases in the experimental group and 21 cases in the control group.  **1.6 Statistical analysis**  Statistical analyses are performed on an intention-to-treat (ITT) basis and included all subjects who are randomized and received at least one treatment after enrollment, using the last observation carried forward (LOCF) method for missing data.  Data are analyzed and processed using SPSS 22.0 statistical software. For demographic indicators and baseline data are used to confirm the distribution of variables using the Shapiro-Wilk test. If the distribution was normal, the measurement data are described as mean± standard deviation (‾ x± s)) to compare the balance between groups, and one-way analysis of covariance (ANCOVA) is used to compare the differences between groups; the count data are described as frequency/percentage, and chi-squared test χ^2^ is used to test the differences; the measurement data that are not normally distributed are expressed as median (upper and lower quartiles), and the Wilcoxon rank-sum test is used to compare the differences between groups. Primary outcome measures are assessed using analysis of covariance (ANCOVA) with baseline values as covariates, secondary outcome measures are assessed using ANCOVA and chi-squared tests, and correlations between the primary outcome and other secondary outcome measures are assessed using Pearson's correlation and Point-Biserial correlation. Statistical analyses are performed using‾ x (95% confidence interval, 95% CI), and all statistical analyses are based on two-tailed hypothesis tests with α=0.05 as the test level.  **1.7 Data collection and management**  ① Data Collection: The Case Report Form (CRF) collection method will be adopted, and the data in the CRF will be entered into the database by the clinical investigator in a timely and accurate manner. Strictly follow the protocol of the study plan to complete the data collection, to ensure that the data collection is accurate, timely and complete.  ② Data management: the inspector verifies the contents of the medical record entry with the original data, the supervisor raises questions based on all the data entered, the participant or researcher answers the questions and corrects the erroneous data, and all records of the corrections are saved in the CRF.  ③ After all data are checked for accuracy, the investigator locks and saves the data.  **2. Technology road map**  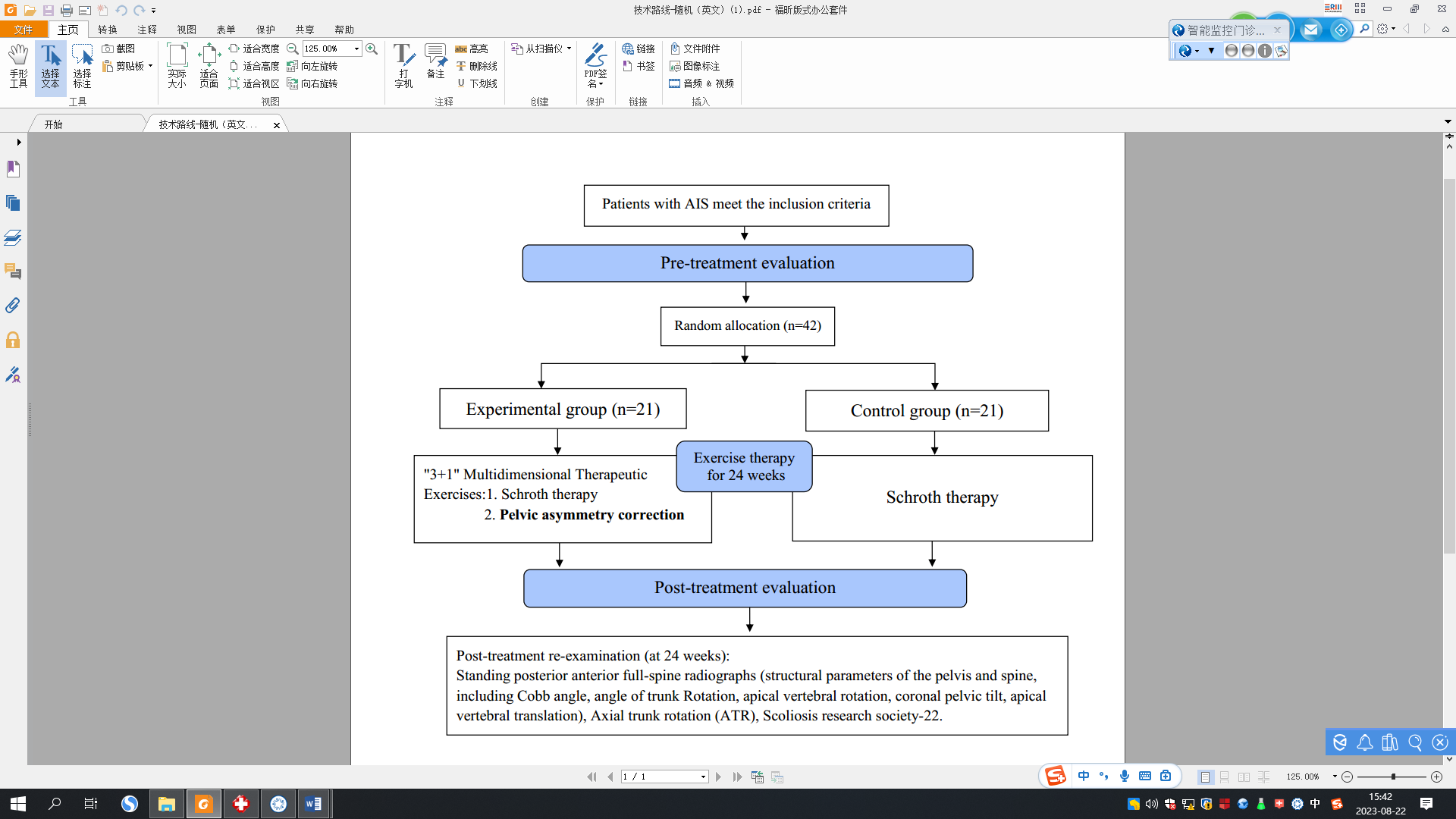  Figure 2. Research technical roadmap  **3. Clinical research quality control measures**  (1) Follow the protocol of the research plan: before the start of the clinical trial, the project director trains the investigators on the protocol of the trial, conducts the trial in an orderly and standardized manner, and completes the treatment in strict accordance with the protocol of the research plan.  (2) Informed Consent Signing: Protect patients' rights and ensure that informed consent is signed in a timely manner.  (3) Adherence to the study program: regular follow-up visits; health education so that parents or guardians play a full role in supervision; maintaining timely communication between therapists and patients and other forms of improving patient adherence.  (4) Quality control of the collection and reporting of all data: training in the process of measuring outcome indicators, with specialized physicians for each indicator; establishment of inspectors to ensure that all elements of the study protocol are strictly adhered to, and monitoring of the original data to ensure consistency with what is reported in the CRF.  (5) Adverse events/reactions: Confirm that all adverse events are recorded and that serious adverse events result in timely management of the patient and that reports are made and recorded within the required timeframe.  (6) Storage and retention of information: Case report information is filed in a timely manner and stored in a dedicated cabinet. |
| --- |
